# Palmitate impairs autophagic degradation via oxidative stress/perilysosomal Ca^2+^ overload/mTORC1 activation pathway in pancreatic **β** cells

**DOI:** 10.1172/jci.insight.192827

**Published:** 2025-11-11

**Authors:** Ha Thu Nguyen, Luong Dai Ly, Thuy Thi Thanh Ngo, Soo Kyung Lee, Carlos Noriega Polo, Subo Lee, Taesic Lee, Seung-Kuy Cha, Xaviera Riani Yasasilka, Kae Won Cho, Myung-Shik Lee, Andreas Wiederkehr, Claes B. Wollheim, Kyu-Sang Park

**Affiliations:** 1 Department of Physiology,; 2 Mitohormesis Research Center,; 3 Department of Global Medical Science, and; 4 Department of Family Medicine, Yonsei University Wonju College of Medicine, Wonju, South Korea.; 5 Soonchunhyang Institute of Medi-bio Science, Soonchunhyang University, Cheonan, South Korea.; 6 Ecole Polytechnique Fédérale de Lausanne, Lausanne, Switzerland.; 7 Department of Cell Physiology and Metabolism, University of Geneva, Geneva, Switzerland.; 8 Department of Clinical Sciences, Lund University, Malmö, Sweden.

**Keywords:** Aging, Endocrinology, Autophagy, Calcium signaling, Diabetes

## Abstract

Saturated fatty acids impose lipotoxic stress on pancreatic β cells, leading to β cell failure and diabetes. In this study, we investigate the critical role of organellar Ca^2+^ disturbance on defective autophagy and β cell lipotoxicity. Palmitate, a saturated fatty acid, induced perilysosomal Ca^2+^ elevation, sustained mTOR complex 1 (mTORC1) activation on the lysosomal membrane, suppression of the lysosomal transient receptor potential mucolipin 1 (TRPML1) channel, and accumulation of undigested autophagosomes in β cells. These Ca^2+^ aberrations with autophagy defects by palmitate were prevented by an mTORC1 inhibitor or a mitochondrial superoxide scavenger. To alleviate perilysosomal Ca^2+^ overload, strategies such as lowering extracellular Ca^2+^, employing voltage-gated Ca^2+^ channel blocker or ATP-sensitive K^+^ channel opener, effectively abrogated mTORC1 activation and preserved autophagy. Furthermore, redirecting perilysosomal Ca^2+^ into the endoplasmic reticulum (ER), with an ER Ca^2+^ ATPase activator, restored TRPML1 activity, promoted autophagic flux, and improved survival of β cells exposed to palmitate-induced lipotoxicity. Our findings suggest oxidative stress/Ca^2+^ overload/mTORC1 pathway involvement in TRPML1 suppression and defective autophagy during β cell lipotoxicity. Restoring perilysosomal Ca^2+^ homeostasis emerges as a promising therapeutic strategy for metabolic diseases.

## Abstract 2

Palmitate triggers ROS-induced Ca^2+ ^overload and mTORC1 activation at the lysosomal membrane, resulting in autophagy defects. Restoring perilysosomal Ca^2+ ^homeostasis offers protection against β-cell lipotoxicity.

## Graphical abstract

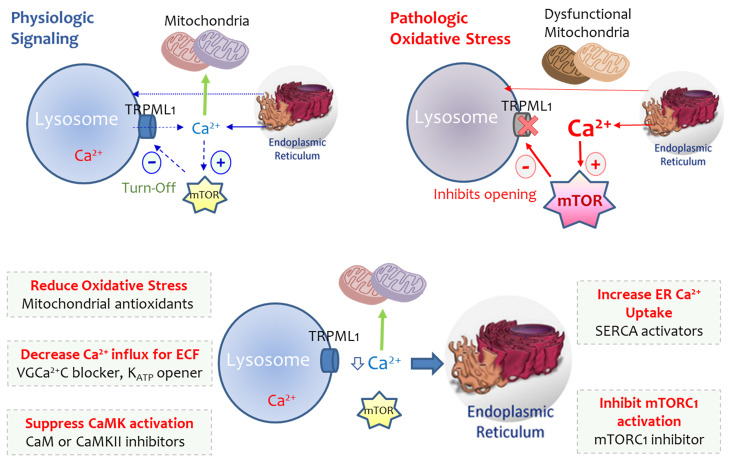

## Introduction

Type 2 diabetes (T2D) presents as a multifaceted metabolic disorder characterized by hyperglycemia resulting from impaired insulin secretion and insulin resistance (1). Overnutrition and physical inactivity precipitate insulin resistance, requiring increased insulin secretion to maintain glucose homeostasis. The capacity to adapt β cell mass and insulin secretion is genetically determined, and β cell decompensation leads to T2D in susceptible individuals (2, 3). Within the diabetic milieu, free fatty acids are recognized as contributors to β cell failure through a process known as lipotoxicity (4). Excess of saturated fatty acids initiates a cellular cascade, including oxidative stress, ER stress, and inflammation, ultimately compromising the functionality and survival of β cells (2).

Growing evidence suggests that impaired autophagic flux is linked to cell death induced by lipotoxicity (5, 6). Autophagy is an evolutionary conserved cellular process responsible for the degradation and recycling of damaged organelles, serving as a vital quality control mechanism to promote cell survival under various stress conditions. Upon lipotoxic stress, autophagy could act as a protective mechanism for β cell survival by eliminating accumulated lipids and damaged organelles. However, chronic exposure to lipid excess, such as in obesity or elevated free fatty acid levels, can lead to an impaired and dysfunctional autophagic machinery (6–8). One potential mechanism by which lipotoxicity impairs autophagy in β cells is through upregulation of mTOR complex 1 (mTORC1) signaling, which negatively regulates autophagic flux. Consistently, suppression of mTORC1 recovered insulin secretion in islets from T2D individuals, which had higher expression level of mTORC1 than those from the controls (9). However, the precise role of chronic mTORC1 activation by lipotoxic stress remains unclear.

Autophagy involves multiple processes, including the initiation, elongation, and maturation of autophagosomes; fusion between autophagosomes and lysosomes; and subsequent degradation of the materials (10). Lipotoxicity has been shown to disrupt the autophagosome-lysosome fusion, which is essential to complete the autophagic process (11–13). This disruption hinders proper degradation, leading to the accumulation of toxic intermediates and damaged organelles. Previous studies suggest that the release of Ca^2+^ from lysosomes can dephosphorylate the transcription factor EB (TFEB), promoting its translocation to the nucleus, where it stimulates the induction of autophagy and lysosome biogenesis. Furthermore, lysosomal Ca^2+^ release triggers the biogenesis of autophagic vesicles through the generation of phosphatidylinositol 3-phosphate (PI3P) and the recruitment of essential PI3P-binding proteins to the nascent phagophore in a TFEB-independent manner (14). Among the various types of Ca^2+^ channels in lysosomes, the mammalian mucolipin subfamily of transient receptor potential (TRPML) channels, also known as mucolipin TRP cation channel 1 (MCOLN), has been identified as particularly critical for autophagic flux (15). Additionally, Ca^2+^ release from the ER or lysosome is required for autophagosome-lysosome fusion, evidenced by the defective vesicular fusion with nonfunctional mutants of MCOLN1 (16) or through thapsigargin treatment (17). It has been shown that mitochondrial ROS directly stimulate TRPML1 to release Ca^2+^. This triggers degradation of dysfunctional ROS-generating mitochondria, a quality control mechanism termed mitophagy (18).

Several studies have clarified the role of cellular Ca^2+^ homeostasis in pancreatic β cell physiology and its dysregulation in lipotoxicity (19). Disturbances in the regulation of ER, mitochondrial, and cytosolic Ca^2+^ due to an excess of saturated fatty acids lead to insulin secretory defects and cytotoxicity (19). In this context, we introduce a potentially novel concept proposing that perilysosomal Ca^2+^ overload in β cells, caused by sustained palmitate exposure, disrupts the proper function of TRPML1-mediated Ca^2+^ transients required for autophago-lysosomal degradation. We investigated molecular mechanisms of organellar Ca^2+^ dysregulation and its detrimental consequences for autophagic flux in palmitate-exposed β cells. In addition, we explored a potentially novel therapeutic strategy aimed to recover perilysosomal Ca^2+^ homeostasis to alleviate lipotoxic stress and preserve β cell function.

## Results

### Palmitate inhibits autophagic flux and causes accumulation of autophagosome in pancreatic islets.

**Figure 1:**
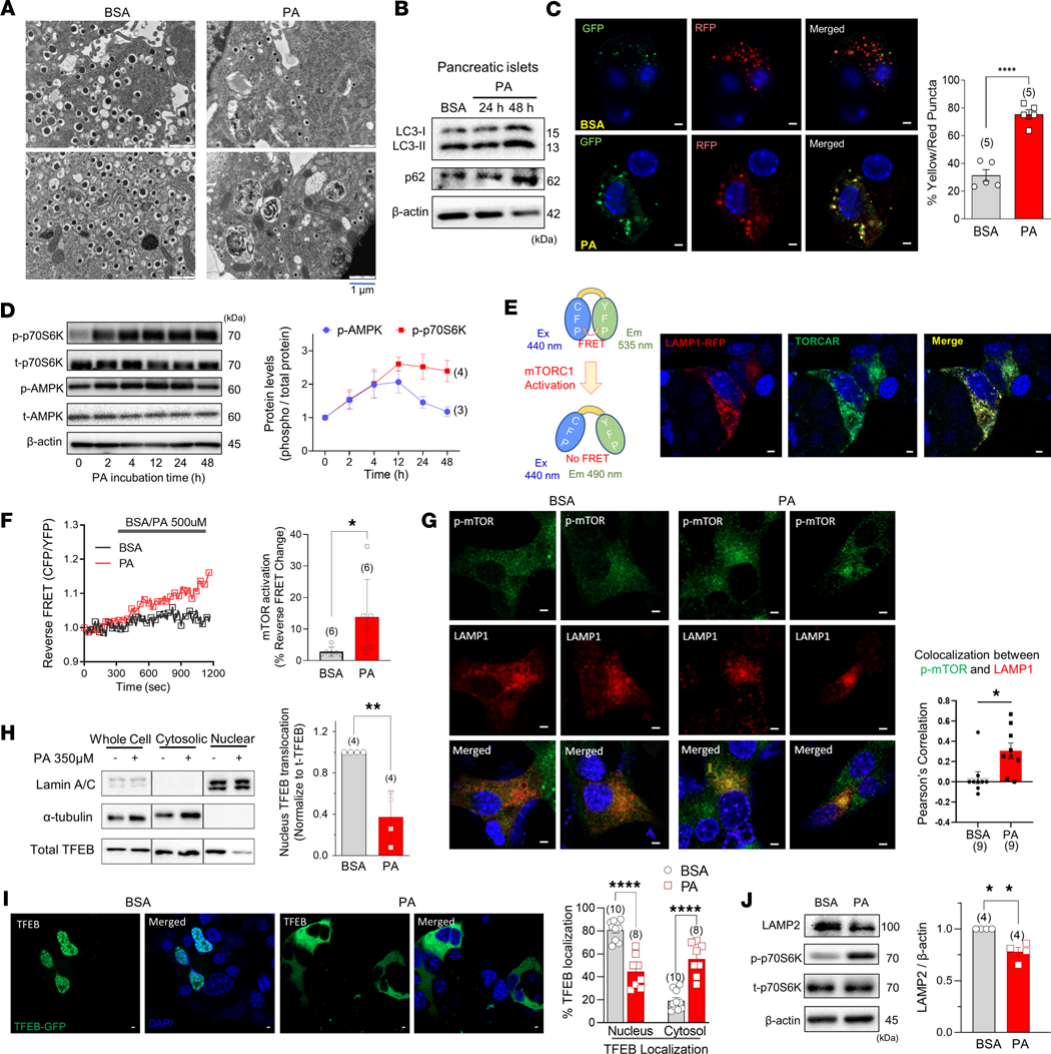
Palmitate induces autophagy defects mediated by mTORC1 activation at lysosomal membranes. (**A**) Representative pictures of electron microscopy showing that exposure to palmitate (PA; 350 μM for 24 hours) causes accumulation of undigested autophagosomes, dilated ER lumen, and reduced number of insulin granules in mouse pancreatic β cells. (**B**) LC3-II and p62 accumulation by PA in pancreatic islets. (**C**) Elevated yellow puncta induced by PA in LC3-GFP-RFP–expressing MIN6 cells. (**D**) Time kinetics of p70S6K and AMPK phosphorylations by PA. (**E**) Lysosomal localization of TORCAR, an indicator of mTORC1 activity. (**F**) TORCAR FRET ratio indicating mTORC1 activity at lysosomes in response to PA. (**G**) Colocalization of phosphorylated (activated) mTOR with LAMP1, a lysosome marker. (**H** and **I**) Decreased nuclear translocation and increased cytosolic localization of TFEB by PA. (**J**) Reduced LAMP2 by PA in MIN6 cells. Scale bar: 1 μm (**C**, **E**, **G**, **I**). Data are presented as means ± SEM (**C**, **D**, **G**–**J**) or SD (**F**), and *n* is the number of independent experiments except those in **H**, which is the number of analyzed cells from at least 3 independent experiments. Statistical significance was determined using unpaired 2-tailed Student’s *t* test (**C** and **F**–**J**). *P < 0.05; **P < 0.01; ****P < 0.0001.

To investigate the impact of saturated fatty acids on autophagic activity in pancreatic β cells, we conducted experiments using isolated mouse islets or MIN6 insulinoma cells treated with palmitate (350 μM). The treatment of islets with palmitate resulted in ER stress, evidenced by dilated ER cisternae, the accumulation of autophagic vesicles, and reduced number of insulin granules, as visualized using transmission electron microscopy (Figure 1A). This accumulation indicated a disruption of autophagy by lipotoxicity in islets. To confirm the defect in autophagy, we examined levels of the autophagic markers LC3 and p62 and the autophagic flux using MIN6 cells stably expressing LC3-RFP-GFP. The protein amounts of LC3-II and p62 were found to be elevated (Figure 1B), and the ratio of yellow to red puncta was increased, indicating defective autophagic flux (Figure 1C).

Since both mTOR and AMPK are known to be master regulators of autophagy, we assessed the activities of mTORC1 and AMPK during palmitate incubation. We observed that palmitate stimulated both AMPK and mTORC1 activity albeit with different kinetics; AMPK phosphorylation was transient, returning to basal within 24 hours, whereas mTORC1 activation was sustained even after 2 days of exposure to lipotoxic conditions (Figure 1D). The continued mTORC1 activation could be responsible for the observed inhibition of autophagy induced by palmitate (2). To monitor the kinetics of mTORC1 activation, we utilized the TORCAR biosensor, a fluorescence resonance energy transfer–based (FRET-based) reporter that detects phosphorylation of 4E-binding protein 1 (4EBP1) (20). TORCAR fluorescence completely colocalized with lysosomal-associated membrane protein 1 (LAMP1), a lysosomal membrane protein (Figure 1E), and palmitate increased reverse FRET ratio of TORCAR, reflecting mTORC1 activity (Figure 1F).

Previous studies have indicated that the activation of mTORC1 occurs when it is recruited to the lysosomal surface in response to an abundance of amino acids (21). Here, we observed that palmitate increased colocalization of activated (phosphorylated) mTORC1 with LAMP1, which suggests activation of mTORC1 on the lysosomal membrane (Figure 1G). Activation of mTORC1 negatively regulates autophagy through various mechanisms, including the inhibition of lysosomal biogenesis by phosphorylating the transcription factor TFEB (22). This phosphorylation leads to the retention of TFEB in the cytosol and hinders its translocation into the nucleus (23). To test whether palmitate inhibits the translocation of TFEB into the nucleus, MIN6 cells were treated with palmitate and examined for the localization of TFEB in cytosolic and nuclear fractions. As shown in Figure 1H, TFEB in the nuclear fraction was significantly reduced by palmitate incubation. Consistent with these findings, transiently expressed TFEB-GFP was retained in the cytosol of palmitate-treated MIN6 cells (Figure 1I). To demonstrate the beneficial role of TFEB against lipotoxicity, MIN6 cells were infected with adenoviruses encoding either wild-type TFEB or an empty vector control. Overexpression of TFEB upregulated LAMP1, restored p62 clearance, and prevented caspase-3 activation in palmitate-treated MIN6 cells (Supplemental Figure 1; supplemental material available online with this article; https://doi.org/10.1172/jci.insight.192827DS1). These findings indicate that palmitate inhibits TFEB activation. As a result, lysosomal biogenesis is impaired as also demonstrated by the reduced expression of the lysosomal marker LAMP2 (Figure 1J).

### Palmitate inhibits TRPML1-mediated lysosomal Ca^2+^ release.

**Figure 2:**
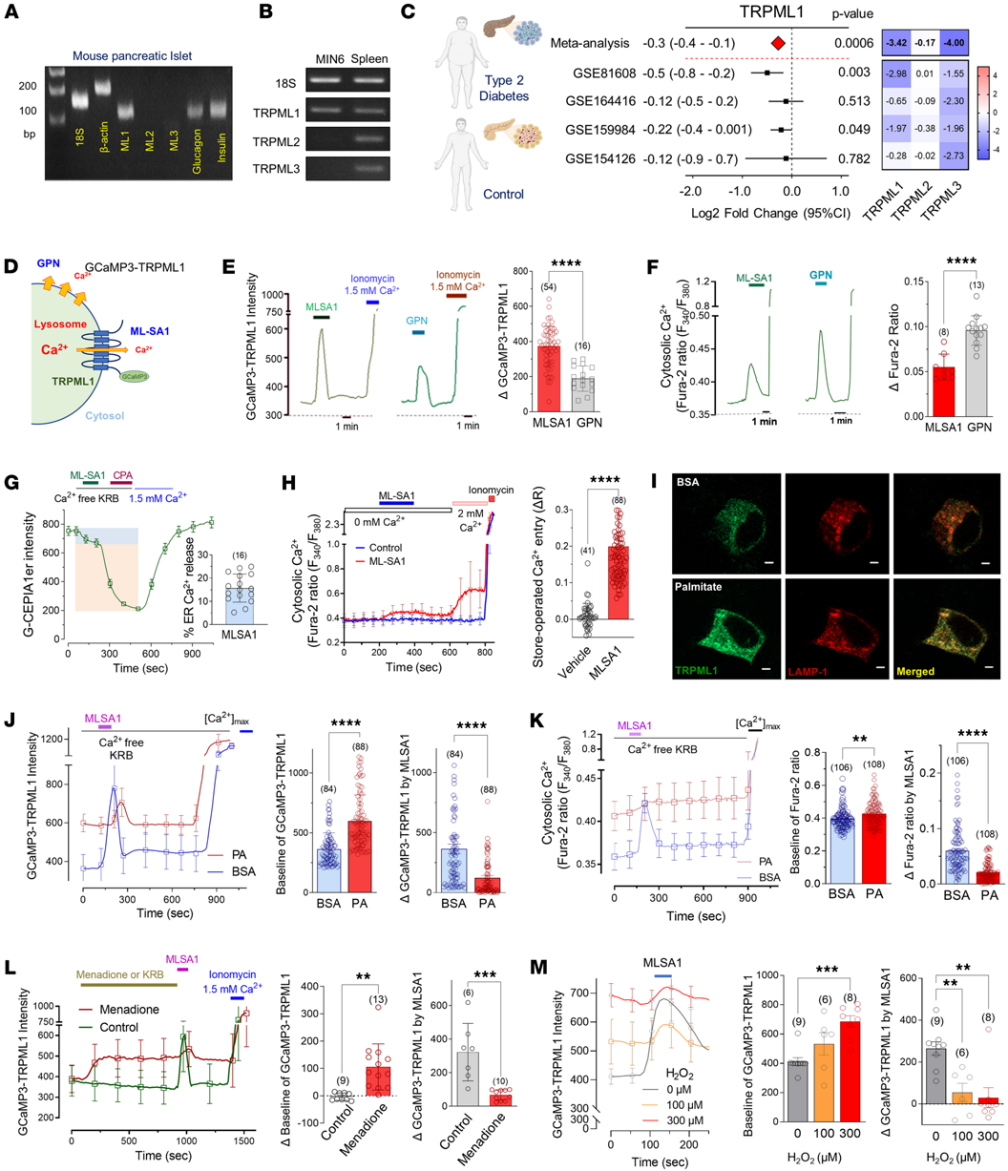
Palmitate elevates perilysosomal Ca^2+^ level and inhibits TRPML1-mediated lysosomal Ca^2+^ release. (**A** and **B**) Expression profile of TRPML channels in mouse pancreatic islets (**A**) and MIN6 cells (**B**). (**C**) Lower expression of TRPML1 in pancreatic islets from patients with type 2 diabetes compared with controls in separate public datasets (black square) and their meta-analyzed fold-change between control and diabetes (red diamond) calculated by Inverse-Variance Weighted method. Heatmaps of TRPMLs show the *z* values (top) calculated by meta-analysis and *t* values from each dataset by DESeq2 analysis. (**D**) GCaMP3-TRPML1 fluorescent probe detecting perilysosomal Ca^2+^ level. (**E** and **F**) MLSA1 (10 μM), a TRPML agonist, and GPN (100 μM), a lysosomotrope, elevated perilysosomal (GCaMP3-TRPML1) and cytosolic (Fura-2) Ca^2+^ levels in MIN6 cells. (**G**) MLSA1 decreased ER Ca^2+^ content measured by G-CEPIA1er, suggesting ER Ca^2+^ release. (**H**) Cytosolic Ca^2+^ rose upon extracellular Ca^2+^ supplementation in MLSA1-treated group, indicating store-operated Ca^2+^ entry. (**I**) Fluorescence imaging of MIN6 cells expressing GCaMP3-TRPML1 treated with BSA or PA. Scale bar: 1 μm (**I**). (**J** and **K**) PA elevated the basal level but abolished MLSA1 response of perilysosomal (**J**) or cytosolic (**K**) Ca^2+^ in MIN6 cells. (**L** and **M**) The oxidants, menadione (100 μM) (**L**) or H_2_O_2_ (**M**), like PA, elevated the baseline Ca^2+^ and abolished the MLSA1 response. [Ca^2+^]_max_ implies cells in normal Ca^2+^ Krebs–Ringer bicarbonate buffer (KRBB) with ionomycin. Data are presented as means ± SD, and (*n*) is the number of analyzed cells from more than 3 independent experiments, except those in **L** and **M** (from 2–3 independent experiments). Statistical significance was determined using unpaired 2-tailed Student’s *t* test (**E**, **F**, **H**, **J**, and **L**) or 1-way ANOVA with post hoc Tukey multiple-comparison test (**M**). **P < 0.01; ***P < 0.001; ****P < 0.0001.

Transient Ca^2+^ increases in the perilysosomal microdomain play a crucial role in the fusion between autophagosomes and lysosomes, a critical step for the degradation of the autophagosome (24). Saturated fatty acids, such as palmitate, induce oxidative stress and disrupt organellar Ca^2+^ homeostasis (19), which may impair the autophagic process because of perilysosomal Ca^2+^ disturbances. To investigate this hypothesis, we analyzed the transcriptional levels of TRPML channels, the primary mediators of Ca^2+^ release from the lysosomal lumen. TRPML1 was the predominant channel expressed in both mouse pancreatic islets (Figure 2A) and MIN6 cells (Figure 2B). To examine the clinical relevance of TRPML1 expression in human islets, we conducted a meta-analysis comparing TRPML1 transcript levels in pancreatic islets from controls and patients with T2D. As shown in the forest plot and heatmap, TRPML1 expression was significantly lower in diabetic islets, similar to the pattern observed for TRPML3, but not for TRPML2 (Figure 2C).

To study Ca^2+^ signaling via TRPML1, we used 3 molecular probes: Fura-2 to investigate cytosolic Ca^2+^, G-CEPIA1er to analyze ER Ca^2+^, and GCaMP3-TRPML1 fusion protein to measure perilysosomal Ca^2+^ levels (Figure 2D). Two specific drugs, MLSA1 (a TRPML1 agonist) and Gly-Phe β-naphthylamide (GPN, a lysosomotropic agent), were utilized to examine lysosomal Ca^2+^ signaling. In cells transfected with GCaMP3-TRPML1, MLSA1 (10 μM) elicited a robust Ca^2+^ signal compared with a lesser increase induced by GPN (100 μM) (Figure 2E). Using Fura-2 to measure changes in cytosolic Ca^2+^, it was observed that MLSA1 induced a smaller increase in total cellular Ca^2+^ level compared with the GPN-induced rise (Figure 2F). Furthermore, previous studies have suggested a close interaction between the ER and lysosomal Ca^2+^, which may be required for replenishing lysosomal Ca^2+^ content from the ER (25, 26). We found that MLSA1-mediated lysosomal Ca^2+^ release decreased the ER Ca^2+^ pool (~15%), suggesting lysosomal Ca^2+^-induced ER Ca^2+^ release (Figure 2G). In response to depletion of the ER Ca^2+^ pool, store-operated Ca^2+^ entry (SOCE) was observed in MLSA1-treated cells. This substantiates the link between ER Ca^2+^ release and TRPML1-mediated lysosomal Ca^2+^ transient (Figure 2H). To explore whether this perilysosomal Ca^2+^ signaling occurs in other cell types, we measured fluorescence changes in human embryonic kidney (HEK) cells stably expressing GCaMP3-TRPML1. Similar to MIN6 cells, MLSA1 induced Ca^2+^ transients in HEK cells, followed by SOCE only in the MLSA1-treated group (Supplemental Figure 2).

To test our hypothesis regarding the effect of palmitate on lysosomal Ca^2+^ signaling, MIN6 cells expressing GCaMP3-TRPML1 were incubated with palmitate or BSA for 24 hours. Using fluorescence microscopy, we observed that palmitate-treated cells exhibited a significant increase in the GCaMP3 fluorescence signal, indicating elevated Ca^2+^ levels in the perilysosomal regions (Figure 2I). Importantly, MIN6 cells incubated with palmitate showed a diminished Ca^2+^ response to MLSA1, suggesting inhibition of Ca^2+^ release via TRPML1 channels (Figure 2J). Furthermore, palmitate-exposed cells displayed an elevated basal cytosolic Ca^2+^ and an attenuated MLSA1-induced Ca^2+^ response (Figure 2K). It has been reported that ROS triggers TRPML1 channel opening (18). To compare the impact of palmitate on lysosomal Ca^2+^ regulation with that of ROS, MIN6 cells expressing GCaMP3-TRPML1 were exposed to the oxidizing agents, menadione (100 μM) or H_2_O_2_ (100 and 300 μM), and perilysosomal Ca^2+^ levels were recorded. Both menadione (Figure 2L) and H_2_O_2_ (Figure 2M) prominently elevated the basal fluorescence intensity, indicating an increase in perilysosomal Ca^2+^ levels. As a consequence, the Ca^2+^ response upon MLSA1 treatment was abrogated. These alterations in lysosomal Ca^2+^ signaling induced by ROS were similar to those caused by palmitate, suggesting that palmitate-induced oxidative stress may contribute to the pathologic dysregulation of lysosome and autophagic defects.

### mTORC1 activation prevents TRPML1-mediated lysosomal Ca^2+^ release and autophagic flux.

**Figure 3:**
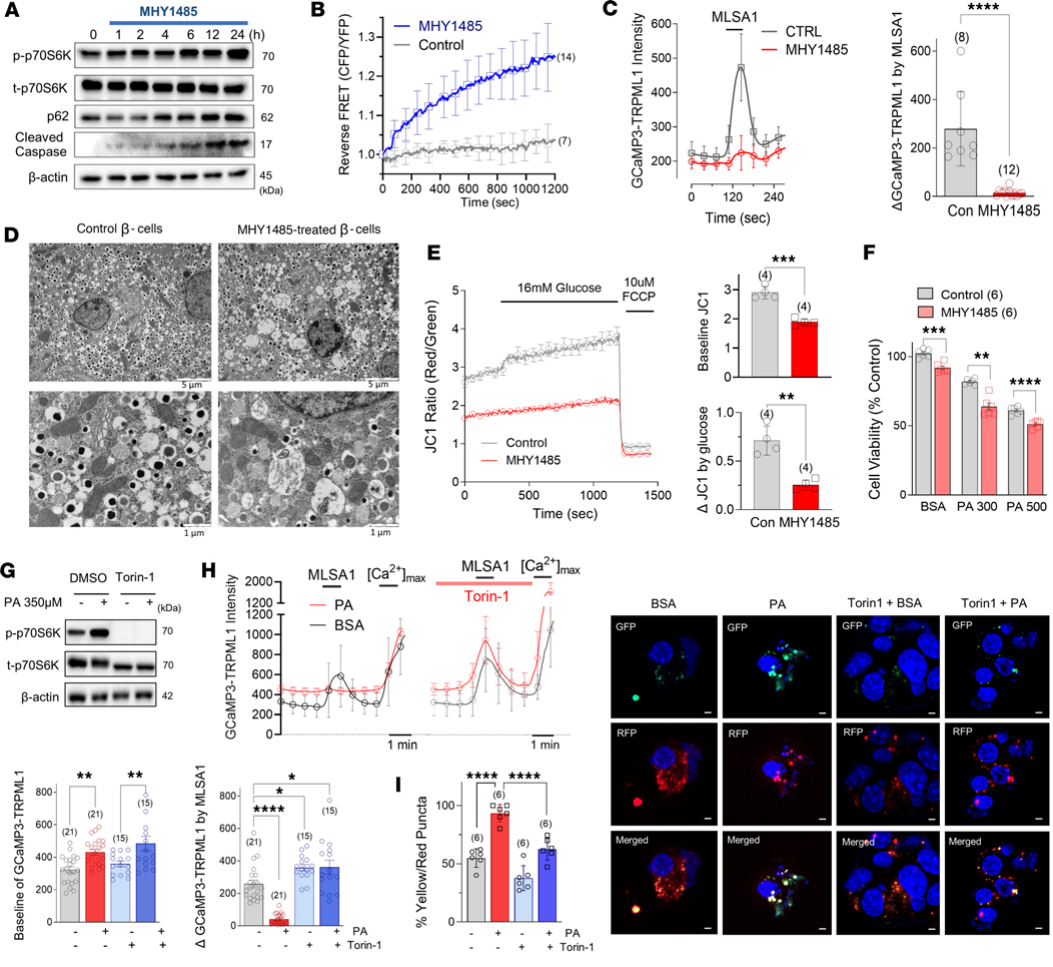
Inhibiting mTORC1 abolishes palmitate-induced TRPML1 suppression and autophagy defects. (**A** and **B**) mTORC1 activator, MHY1485 (10 μM), augmented mTORC1 signaling and increased p62 and cleaved caspase-3. (**C**) Pharmacologic activation using MYH1485 inhibited MLSA1-triggered lysosomal Ca^2+^ release in MIN6 cells. (**D** and **E**) mTOR activator caused accumulation of undigested autophagosomes and depolarized mitochondrial membrane potential, leading to impaired glucose responses measured by JC1 fluorescence. (**F**) The mTOR activator, MYH1485, aggravated PA-induced cell death measured by using MTT assay. (**G** and **H**) Torin-1 (100 nM), an mTORC1 inhibitor, reduced basal perilysosomal Ca^2+^ level but recovered MLSA1-triggered lysosomal Ca^2+^ release. (**I**) Torin-1 recovered PA-increased yellow/red puncta in LC3-GFP-RFP–expressing cells. Scale bar: 1 μm (**I**). Data are presented as means ± SD (**B**, **C**, and **H**) or SEM (**E** and **F**), and *n* is the number of analyzed wells or cells from more than 3 independent experiments. Statistical significance was determined using unpaired 2-tailed Student’s *t* test (**C**, **E**, and **F**) or 1-way ANOVA with post hoc Tukey multiple-comparison test (**H** and **I**). *P < 0.05; **P < 0.01; ***P < 0.001; ****P < 0.0001.

As shown in Figure 1, palmitate activated mTORC1 located at the lysosomal membrane, interfering with nuclear TFEB translocation and lysosomal biogenesis. To understand the molecular mechanisms of mTORC1-mediated autophagy defects, we utilized MHY1485, an mTORC1 activator. As expected, MHY1485 (10 μM) induced phosphorylation of S6K, accumulation of the autophagic marker p62, and increased cleaved caspase-3 in a time-dependent manner (Figure 3A). Additionally, the mTORC1 activity reporter TORCAR detected a marked increase in mTOR activity upon MHY1485 treatment (Figure 3B). Intriguingly, MHY1485 incubation for 12 hours almost completely suppressed the MLSA1-induced perilysosomal Ca^2+^ response (Figure 3C), consistent with palmitate-suppressed TRPML1 activity (Figure 2J). Furthermore, when mouse islets were cultured with MHY1485, we observed enlarged ER lumen and the accumulation of autophagic vesicles (Figure 3D), similar to the phenotype of palmitate-treated islets (Figure 1A). Excessive mTORC1 activation by MHY1485 also led to mitochondrial membrane depolarization in MIN6 cells (Figure 3E). Coincubation of palmitate with MHY1485 resulted in aggravated lipotoxicity in MIN6 cells, highlighting the detrimental consequences of mTORC1 activation in lipotoxicity (Figure 3F).

To investigate the putative therapeutic value, we employed the specific mTORC1 inhibitor, Torin-1 (100 nM), which completely abolished S6K phosphorylation (Figure 3G). Torin-1 restored perilysosomal Ca^2+^ signaling blunted by palmitate (Figure 3H). Additionally, using LC3-RFP-GFP, we observed that the inhibition of mTORC1 recovered autophagic flux in palmitate-treated cells (Figure 3I). Collectively, these findings provide evidence for the negative influence of mTORC1 activation induced by palmitate incubation on TRPML1 activity and its implications in autophagy and lipotoxicity.

### Scavenging mitochondrial ROS relieves palmitate-induced autophagic defects.

**Figure 4:**
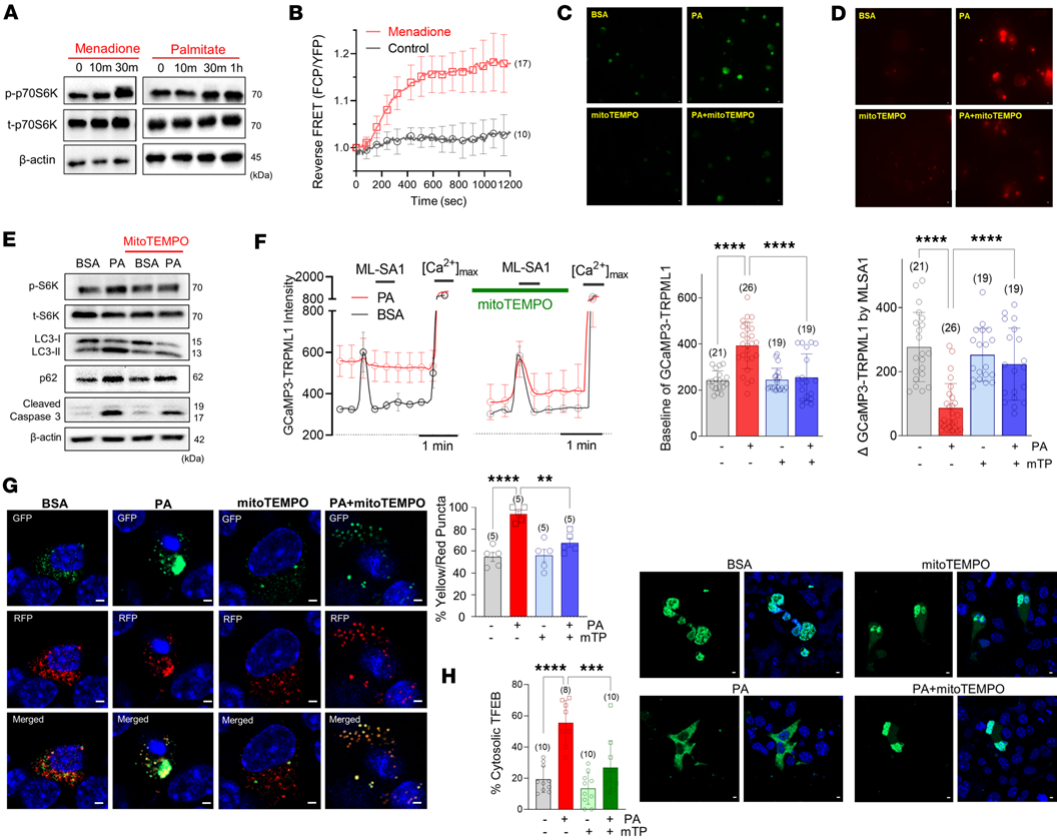
Scavenging mitochondrial superoxide prevents palmitate-induced TRPML1 inhibition and autophagy defects. (**A** and **B**) Menadione, an oxidant, activated mTORC1 signaling. (**C** and **D**) Reduction of cytosolic (DCF) and mitochondrial (mitoSox) ROS by the mitochondrial superoxide scavenger, mitoTEMPO (mTP, 100 nM). (**E**) MitoTEMPO alleviated PA-increased LC3-II, p62, activated mTOR, and cleaved caspase-3. (**F**) MitoTEMPO prevented perilysosomal Ca^2+^ elevation and restored the MLSA1 response attenuated by PA. (**G**) MitoTEMPO restored PA-induced defective autophagic degradation measured in LC3-GFP-RFP–expressing MIN6 cells. (**H**) MitoTEMPO counteracted PA-induced cytosolic retention of TFEB. Scale bar: 1 μm (**C**, **D**, **G**, **H**). Data are presented as means ± SEM (**G** and **H**) or SDs (**B** and **F**). *n* is the number of independent experiments (**G** and **H**) or analyzed cells (**B** and **F**) from more than 3 independent experiments. Statistical significance was determined using 1-way ANOVA with post hoc Tukey multiple-comparison test (**F**–**H**). **P < 0.01; ***P < 0.001; ****P < 0.0001.

In the previous section, we presented the association between oxidative stress and elevated perilysosomal Ca^2+^ levels, as well as the impaired response to MLSA1 (Figure 2, L and M). To examine the potential impact of increased ROS on mTORC1 activation, we applied oxidative stress by exposing MIN6 cells to menadione (100 μM) and assessed mTORC1 activation. Menadione strongly activated mTORC1 as demonstrated by phosphorylation of S6K (Figure 4A). Menadione also increased the reverse FRET ratio of TORCAR (Figure 4B). To study the role of mitochondrial ROS in palmitate-induced oxidative stress, we incubated dispersed mouse islet cells with mitoTEMPO (100 nM), a mitochondrial superoxide scavenger. The application of mitoTEMPO substantially reduced both cytosolic and mitochondrial ROS levels, as measured by DCF and mitoSox, respectively (Figure 4, C and D). Western blot analysis further revealed that mitoTEMPO blunted palmitate-induced S6K phosphorylation, LC3-II and p62 accumulation, as well as caspase activation (Figure 4E). Importantly, scavenging mitochondrial superoxide with mitoTEMPO prevented palmitate-induced perilysosomal Ca^2+^ dysregulation. mitoTEMPO lowered basal Ca^2+^ levels and recovered MLSA1-induced perilysosomal Ca^2+^ rises (Figure 4F). Furthermore, mitoTEMPO promoted TFEB translocation to the nucleus, rescuing TFEB from palmitate-induced retention in the cytosol (Figure 4H). As a consequence of the normalization of lysosomal Ca^2+^ signaling, autophagic flux was restored, as measured using LC3-GFP-RFP processing (Figure 4G). These findings provide evidence that mitochondrial oxidative stress caused by palmitate induces mTORC1-mediated perilysosomal Ca^2+^ dysregulation and autophagic defects.

### Preventing Ca^2+^ overload alleviates palmitate-induced mTORC1 activation and autophagic blockage.

**Figure 5:**
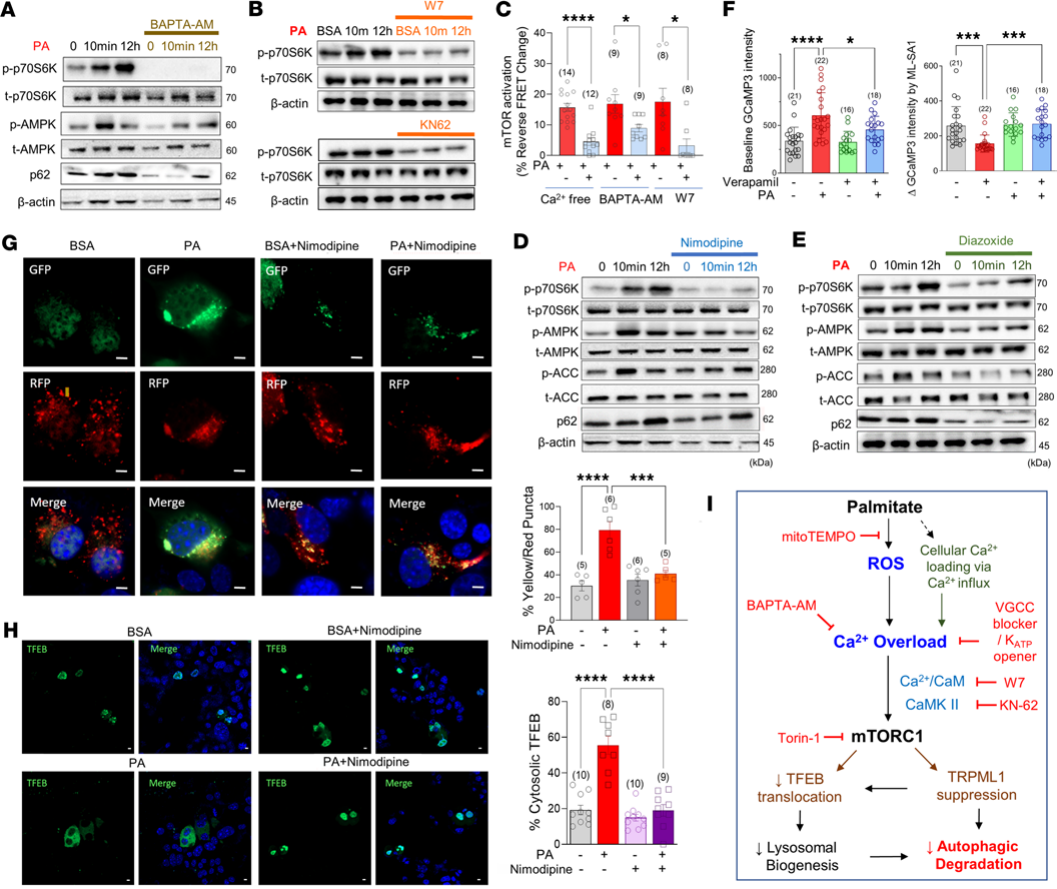
Reducing Ca^2+^ influx attenuates palmitate-induced mTORC1 activation and autophagy defects. (**A**) Intracellular Ca^2+^ chelation using BAPTA-AM inhibited PA-induced p70S6K and AMPK activation. (**B**) Inhibitors of calmodulin (W7, 10 μM) and CaMKII (KN-62, 20 μM) prevented PA-activated p70S6K. (**C**) PA-induced mTORC1 activation was abrogated by extracellular Ca^2+^-free medium, BAPTA-AM (10 μM), or W7 measured in TORCAR-expressing MIN6 cells. (**D** and **E**) PA-induced activation of p70S6K and AMPK was abolished by nimodipine (**D**, 10 μM), a VGCC inhibitor, or diazoxide (**E**, 200 μM), an ATP-sensitive K^+^ channel opener. (**F**) PA effects on perilysosomal Ca^2+^ level and MLSA1 responses were prevented by a voltage-gated Ca^2+^ channel (VGCC) inhibitor, verapamil (10 μM). (**G**) Nimodipine restored nuclear localization of TFEB. (**H**) PA-induced defective autophagic degradation was reestablished by nimodipine, measured in LC3-GFP-RFP–expressing cells. Scale bar: 1 μm (**G**, **H**). (**I**) Hypothetical mechanisms of autophagic defects in PA-induced β cell lipotoxicity. Data are presented as means ± SEM (**G** and **H**) or SDs (**C** and **F**). *n* is the number of independent experiments (**G** and **H**) or analyzed cells (**C** and **F**) from more than 3 independent experiments. Statistical significance was determined using unpaired 2-tailed Student’s *t* test (**C**) or 1-way ANOVA with post hoc Tukey multiple-comparison test (**F**–**H**). *P < 0.05; ***P < 0.001; ****P < 0.0001.

We have shown above that oxidative stress elicits strong mTORC1 activation (Figure 3, A and B), which leads to detrimental consequences on lysosomal Ca^2+^ signaling and autophagic flux. However, the molecular connection between oxidative stress and mTORC1 activation remains unclear. Notably, it has been reported that amino acid–induced mTORC1 activation at the lysosomal membrane is dependent on Ca^2+^/calmodulin (CaM) (27). To understand the pathogenic role of cellular Ca^2+^ dysregulation by palmitate, MIN6 cells were subjected to different conditions, including nominally Ca^2+^-free medium and intracellular Ca^2+^ chelation with BAPTA-AM (10 μM). To further investigate the downstream signaling, we also used W7 (10 μM) and KN62 (20 μM) to inhibit CaM and CaM kinase II (CaMKII), respectively. All conditions with lowered cytosolic Ca^2+^ level or CaM/CaMKII inhibition abolished palmitate-induced mTORC1 activation, indicating the key role of Ca^2+^ and CaM in the activation of mTORC1 during lipotoxicity (Figure 5, A–C).

During β cell metabolic activation, ATP-sensitive K^+^ (K_ATP_) channels close, causing plasma membrane depolarization. As a consequence, voltage-gated Ca^2+^ channels (VGCCs) open and act as the primary Ca^2+^ influx mechanism triggering insulin secretion. To evaluate the impact of extracellular Ca^2+^ influx on palmitate-induced mTORC1 activation and autophagy suppression, MIN6 cells were incubated with either the VGCC blockers, verapamil (10 μM) and nimodipine (10 μM), or the K_ATP_ channel opener, diazoxide (200 μM). Diazoxide induces strong hyperpolarization and prevents opening of VGCCs in insulin-secreting β cells. Not only VGCC blockade but also K_ATP_ channel opening abrogated palmitate-induced mTORC1 activation and p62 accumulation, further verifying the critical role of VGCCs in palmitate-induced Ca^2+^ overload (Figure 5, D and E). Verapamil recovered perilysosomal Ca^2+^ homeostasis, reduced basal Ca^2+^ level, and restored the Ca^2+^ response to MLSA1 (Figure 5F). This is consistent with our previous report about verapamil-induced suppression of oxidative stress and lipotoxicity (28). Moreover, inhibition of VGCC-mediated Ca^2+^ influx preserved autophagic flux under palmitate toxicity in MIN6 cells (Figure 5G) and restored the nuclear localization of TFEB (Figure 5H). Based on our findings, a molecular mechanism is proposed in which palmitate-induced cytosolic Ca^2+^ overload requires extracellular Ca^2+^ influx via VGCCs, which in turn activates mTORC1 through the involvement of CaM/CaMKII. mTORC1 activation suppresses TRPML1 activity and decreases TFEB translocation into the nucleus, ultimately contributing to the blockage of autophagy during lipotoxicity (Figure 5I).

### Activating the SERCA relieves palmitate-induced Ca^2+^ overload and rescues autophagic activity.

**Figure 6:**
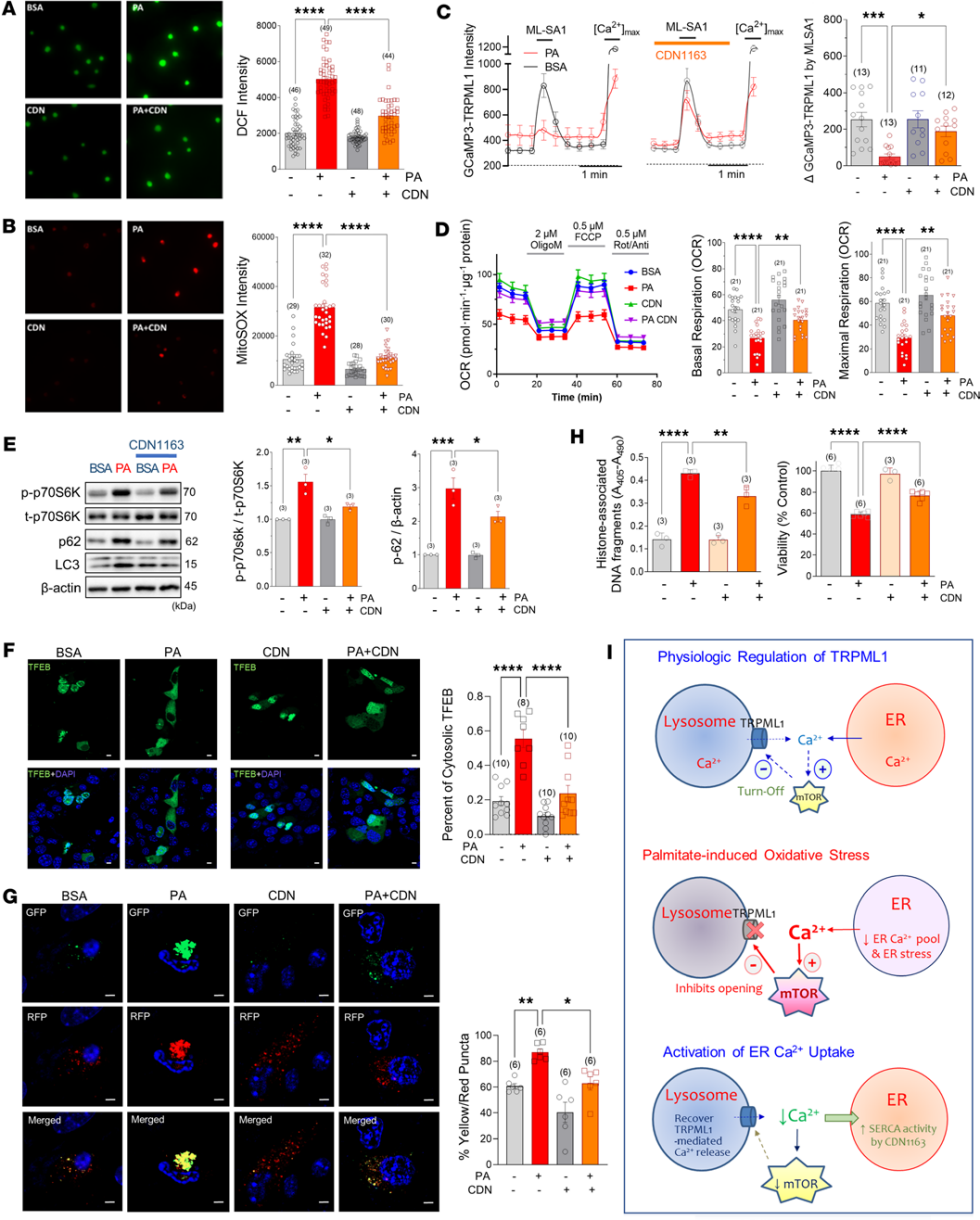
Activating ER Ca^2+^ uptake recovers perilysosomal Ca^2+^ homeostasis and autophagy activity. (**A** and **B**) CDN1163 (10 μM) abrogated PA-induced cytosolic (DCF; **A**) and mitochondrial (mitoSOX; **B**) ROS generation. (**C**) CDN1163 prevented PA effects on perilysosomal Ca^2+^ level and MLSA1 responses. (**D**) CDN1163 recovered basal and FCCP-induced maximal oxygen consumption. FCCP, carbonyl cyanide *p*-trifluoromethoxyphenylhydrazone; OCR, oxygen consumption rate. (**E**) CDN1163 attenuated p70S6K activation and p62 accumulation. (**F**) CDN1163 prevented the inhibition of nuclear localization of TFEB by PA. (**G**) PA-induced defective autophagic degradation was recovered by CDN1163, measured in LC3-GFP-RFP–expressing cells. (**H**) CDN1163 decreased PA cytotoxicity, measured by cell viability (MTT) and apoptotic (cell death detection) assays in MIN6 cells. (**I**) Schematic hypothesis of palmitate-induced TRPML1 suppression and its recovery by activating ER Ca^2+^ uptake. Scale bars: 1 μm. Data are presented as means ± SEM (**C**–**H**) or SDs (**A** and **B**). *n* is the number of independent experiments (**C**–**H**) or analyzed cells (**A** and **B**) from more than 3 independent experiments. Statistical significance was determined using 1-way ANOVA with post hoc Tukey multiple-comparison test (**A**–**H**). *P < 0.05; **P < 0.01; ***P < 0.001; ****P < 0.0001.

ER Ca^2+^ depletion is a primary mechanism responsible for inducing ER stress and cytosolic Ca^2+^ overload in lipotoxicity. Multiple mechanisms contribute to the depletion of ER Ca^2+^ stores, and one of them is impaired smooth endo/sarcoplasmic Ca^2+^ ATPase (SERCA) activity (19). To probe for SERCA implication, we utilized CDN1163 (10 μM), a SERCA pump activator, to investigate whether enhancing ER Ca^2+^ uptake through the SERCA pump could rescue autophagic impairment and cell death. To follow ER Ca^2+^ depletion, we monitored cytosolic Ca^2+^ increases triggered by SERCA pump inhibition using cyclopiazonic acid (20 μM). The incubation of cells with palmitate significantly diminished the content of ER Ca^2+^, while cotreatment with CDN1163 restored the ER Ca^2+^ pool depleted by palmitate (Supplemental Figure 3), which is consistent with our previous report (29). Furthermore, along with alleviating of ER stress, this SERCA activator reduced cytosolic and mitochondrial ROS generation caused by palmitate (Figure 6, A and B). Palmitate-induced elevation of basal perilysosomal Ca^2+^ levels and inhibition of MLSA1 responses were alleviated by cotreatment with CDN1163 (Figure 6C). Moreover, CDN1163 recovered palmitate-induced suppression of basal and maximal mitochondrial respiration, suggesting improvement of mitochondrial functions by activating ER Ca^2+^ uptake (Figure 6D). CDN1163 alleviated mTORC1 activation and p62 as well as LC3-II accumulation induced by palmitate (Figure 6E). Likewise, CDN1163 facilitated the translocation of TFEB into the nucleus and restored autophagic flux (Figure 6, F and G). Consequently, CDN1163 inhibited cell death induced by palmitate in MIN6 cells (Figure 6H). Furthermore, CDN1163 attenuated palmitate-induced lipid peroxidation associated with ferroptosis, indicating its potential role in the cell death pathway of lipotoxicity (Supplemental Figure 4). By augmenting the activity of the SERCA pump, the uptake of Ca^2+^ into the ER lumen can restore ER Ca^2+^ levels and recover perilysosomal Ca^2+^ overload induced by palmitate. These actions could exert a beneficial effect on autophagic turnover by relieving cytosolic Ca^2+^ overload and ROS generation, which in turn mitigates mTORC1-mediated TRPML1 suppression in β cells (Figure 6I).

## Discussion

In the present study, we elucidated the mechanism underlying palmitate-evoked inhibition of autophagic flux in pancreatic β cells. Importantly, palmitate induces prolonged activation of mTORC1 at the lysosomal membrane, which is driven by elevated perilysosomal Ca^2+^ levels. Dysregulated mTORC1 activation caused by palmitate disrupts physiologic Ca^2+^ conductance through lysosomal Ca^2+^ channels and prevents the nuclear translocation of TFEB. These disturbances result in substantial pathogenic consequences that contribute to the blockage of autophagy. Our findings provide important insights into the critical role of oxidative stress/Ca^2+^ overload/mTORC1-linked cytopathology underlying the autophagic defects observed in pancreatic β cells subjected to lipotoxicity.

Our previous investigations had focused on the depletion of ER Ca^2+^ and mitochondrial dysfunction associated with palmitate-induced oxidative stress (19, 30). To attenuate mitochondrial superoxide production, we aimed to reduce the expression of mitochondrial Ca^2+^ uniporter but observed palmitate-induced cytosolic Ca^2+^ overload and aggravation of autophagy blockage (28). These findings were corroborated by electron micrographic analyses, clearly showing extensive accumulation of undigested autophagosome-like vesicles in palmitate-incubated islets, which prompted the current study to understand the Ca^2+^ dynamics and homeostasis in inter-organellar ionic microdomains. We observed the retention of LC3-II and p62, as well as the downregulation of lysosomal biogenesis and function by palmitate, underlining the importance of autophagosome degradation in β cell lipotoxicity. To elucidate the molecular connection between palmitate and autophagic defects, we investigated the main regulatory signaling pathways in autophagy. Our results revealed that palmitate treatment activates the two antagonistic signaling events, AMPK and mTORC1, with different kinetics. AMPK activation was transient, whereas mTORC1 was sustained for an extended duration. The activity of mTORC1 is known to impede various phases of the autophagic process, through the regulation of key components such as ULK1 (31), PIK3C3 (32), AMBRA1 (33), UVRAG (34), and TFEB (23). Thereby, activation of mTORC1 by palmitate ultimately leads to impaired autophagy and worsened cell fate. We also observed that palmitate markedly lowered the nuclear translocation of TFEB, possibly caused by mTORC1-mediated phosphorylation.

An intriguing observation from public datasets is that the transcript levels of the Ca^2+^ channels TRPML1 and TRPML3 are lower in pancreatic islets from patients with T2D. Alterations in lysosomal Ca^2+^ channel expression and function in human diabetes pathogenesis has not been studied previously. In pancreatic islets, as well as MIN6 cells, the main isotype of TRPML channel was TRPML1 expressed in lysosomes. Of note, TRPML3 is less active at acidic pH and known to play a more prominent role in early endosomes (35). We investigated the role of the TRPML1 channel in organellar Ca^2+^ dynamics in β cells and demonstrated that ER Ca^2+^ reservoir is utilized by the stimulation of TRPML1-mediated lysosomal Ca^2+^ release (Figure 2G). This was further supported by the activation of SOCE resulting from ER Ca^2+^ depletion (Figure 2H). Our findings are consistent with earlier reports that observed ER Ca^2+^ release (CICR) in response to Ca^2+^ release from lysosomes (36, 37). Two possible functional implications of CICR between lysosome and ER can be proposed. First, it has been suggested that the lysosomal Ca^2+^ pool may be replenished from the ER Ca^2+^ through physical contacts mediated by IP_3_ receptors. This ER-to-lysosome Ca^2+^ transfer is crucial for maintaining lysosomal Ca^2+^ homeostasis as well as for the autophagy process. Alternatively, the release of ER Ca^2+^ into the perilysosomal region may amplify the lysosomal Ca^2+^ signal, which is necessary for triggering the fusion between autophagosome and lysosome, thereby facilitating autophagic flux. To better understand these processes, further elucidation of the machineries involved in CICR is needed.

We have positioned the sustained activation of mTORC1 as a key mechanism linking lipotoxicity to autophagy defects. It has been proposed that TRPML1 and mTORC1 regulate each other through a feedback loop, which could enable transient regulation of TRPML1 during physiological stimulation (38). Our results show that prolonged pharmacological activation (Figure 3C) or chronic stimulation of mTORC1 during lipotoxicity (Figure 2J) negatively affects TRPML1 channel. Moreover, the detrimental consequences of pharmacological mTORC1 activation, including the accumulation of autophagosome, mitochondrial dysfunction, and cell death (Figure 3, C and D), are qualitatively similar to those caused by palmitate (Figure 1). As anticipated, inhibition of mTORC1 using Torin-1 successfully restored TRPML1 activity and autophagic flux (Figure 3, H and I). Based on this evidence, we propose that mTORC1 could be a drug target to restore autophagic flux in β cells suffering from lipotoxic stress.

We then elucidated the mechanisms linking lipotoxicity to mTORC1 activation. We observed that strong oxidants, such as menadione and H_2_O_2_, elevated the basal perilysosomal Ca^2+^ levels, which may originate from both lysosomes and the ER. This elevation subsequently suppressed TRPML1 activity and the Ca^2+^ transient induced by MLSA1. Additionally, both oxidant agents and palmitate were found to induce sustained activation of mTORC1. Based on our findings, we postulate that palmitate-induced oxidative stress increases Ca^2+^ release into the perilysosomal microdomain, where it is crucial for mTORC1 activation. This scenario was further validated by MitoTEMPO, a mitochondrial ROS scavenger, which effectively abrogated perilysosomal Ca^2+^ elevation and restored TRPML1 activity, TFEB nuclear translocation, and autophagic degradation in palmitate-treated cells. Oxidative stress is therefore likely causing perilysosomal Ca^2+^ overload, resulting in mTORC1 overstimulation. Previous studies have indicated that mTORC1 stimulation by nutrients, particularly amino acids, is mediated by Ca^2+^/CaM signaling, which is preceded by its recruitment to the lysosomal membrane (21, 27). Similar mechanisms may be responsible during palmitate-induced Ca^2+^ signaling, as demonstrated by our finding that palmitate activates mTORC1 on the lysosomal membrane (Figure 1G) that was abolished by intracellular Ca^2+^ chelation using BAPTA-AM (Figure 5A). Furthermore, we demonstrated that inhibitors of CaM (W7) or CaMKII (KN62) abolished mTORC1 activation induced by palmitate (Figure 5B), suggesting a Ca^2+^/CaM-dependent mTORC1 activation under lipotoxic conditions. It has been proposed that cytosolic Ca^2+^ overload is a crucial mechanism underlying β cell glucolipotoxicity (39). An uncontrolled increase in cytoplasmic Ca^2+^ has been known to trigger several deleterious effects, including activation of protein kinase C, c-Jun N-terminal kinase, C/EBP homologous protein, calpains, and caspases, ultimately leading to mitochondrial dysfunction, ER stress, and apoptosis (40, 41). Here, we propose another mechanism that sustained activation of mTOR and the resultant defective autophagy is an important part of the cytotoxic mechanisms associated with Ca^2+^ overload.

In order to reduce cellular Ca^2+^ content preventing Ca^2+^ overload, we applied different strategies, including the direct inhibition of VGCCs and the prevention of their opening through hyperpolarization, both of which decrease extracellular Ca^2+^ influx (42). Consistent with the effects observed with BAPTA-AM treatment, the use of VGCCs’ blockers or K_ATP_ opener prevented mTORC1 activation and autophagy defects in the presence of palmitate. This suggests that counteracting cytosolic Ca^2+^ load holds promising therapeutic potential for mitigating β cell lipotoxicity. Oxidative stress in the ER activates the release of Ca^2+^ from the ER and inhibits active Ca^2+^ reuptake via SERCA. We demonstrated that CDN1163, a SERCA activator, rescued pancreatic β cells from lipotoxicity by enhancing ER Ca^2+^ uptake (29). In this investigation, we established that promoting ER Ca^2+^ uptake recovered intracellular Ca^2+^ homeostasis, improved mitochondrial function as well as glucose-stimulated insulin secretion, and ultimately rescued cells from lipotoxicity-induced death. Additionally, CDN1163 restored autophagic flux in β cells, along with the normalization of perilysosomal Ca^2+^ levels and TRPML1-mediated Ca^2+^ transients. These findings support the notion that promoting ER Ca^2+^ uptake can alleviate cytosolic Ca^2+^ overload, downregulate mTORC1 signaling, and restore autophagy in palmitate-induced lipotoxicity. However, excessively high ER Ca^2+^ levels may induce mitochondrial Ca^2+^ overload and apoptosis (43), though such effects were not observed in CDN1163-treated cells (Supplemental Figure 5). Our data highlight the potential interplay between oxidative stress, Ca^2+^ dysregulation, and mTORC1 activation in the context of TRPML1 inhibition and defective autophagic degradation in β cell lipotoxicity, as illustrated in Figure 5I and Figure 6I.

Collectively, our study identifies a potentially novel ROS/Ca^2+^ overload/mTORC1 cytopathic mechanism that underlies impaired autophagy and proposes various protective strategies to correct or relieve pathogenic alteration in β cell lipotoxicity. Prolonged activation of mTORC1 by saturated fatty acids plays a critical role in inhibiting autophagy, as it suppresses TRPML1 activity and diminishes the nuclear localization of TFEB. Notably, reducing cellular Ca^2+^ loads by inhibiting extracellular Ca^2+^ influx or promoting Ca^2+^ reuptake into the ER could serve as efficient therapeutic strategies to alleviate oxidative stress and counteract uncontrolled mTOR activation. These results contribute to enhance our understanding of the mechanisms behind lipotoxicity-induced autophagic inhibition and may inform the development of therapeutic interventions aimed at restoring autophagy and preserving pancreatic β cell function in lipotoxic states. Investigations into other pathological conditions characterized by the accumulation of undigested autophagosomes could broaden the applicability of the therapeutic strategies suggested by our results.

## Methods

### Sex as a biological variable.

Our study examined male mice due to less variability in phenotype. It is unknown whether the findings are relevant for female mice.

### Reagents and tools.

All information is described in Supplemental Table 1.

### Cell culture.

MIN6 cells (C0018008, AddexBio Technologies) were cultivated in a controlled environment with 5% CO_2_ at 37°C, using Dulbecco’s modified Eagle medium (DMEM) supplemented with 12 mmol/L glucose. The culture medium consisted of fetal bovine serum (10%), β-mercaptoethanol (5 μL/L), streptomycin (100 μg/mL), and penicillin (100 U/mL) obtained from Hyclone, Thermo Fisher Scientific. Passages ranging from 27 to 39 were selected for our investigation. The medium was refreshed every 2 days, and cells were passaged once a week following detachment using trypsin-EDTA. Prior to conducting experiments, the MIN6 cells were allowed to settle and cultured for 12–24 hours.

### Mouse islet isolation.

All animal care and procedures were performed in accordance with the policies and approval (YWC-200907-2) of the Institutional Animal Care and Use Committee of Yonsei University Wonju College of Medicine. Male 10- to 12-week-old C57BL/6N mice (from DBL) were sacrificed for islet isolation.

A C57BL/6N male mouse was sacrificed by cervical dislocation after general anesthesia. After disinfection by 70% ethanol, the mouse was laid on the tray with tail down. A triangle was incised on the skin starting from the tail. Skin was rolled up and pulled up over the head of sternum, and then hemostatic forceps were clamped at the sternum to keep the abdominal cavity opened. Guts and organs were displaced to the right side and the common bile duct was uncovered. By tracking along the common bile duct, ampulla of Vater could be found. It appeared as a white spot and was clamped by hemostatic forceps. The 2 forceps were perpendicular. The common bile duct was then separated from vessels and liver and was catheterized using a 5 mL syringe with 30.5G needle. About 3 mL of collagenase solution was injected into the common bile duct, distending the pancreas. Dressing forceps were used to detach guts from pancreas. The pancreas was cut off from spleen and was quickly put into a 50 mL conical tube with 5 mL Collagenase (C9263 MilliporeSigma) on ice.

The water bath (37°C) and cool centrifugation machine (4°C) were turned on 1 hour before the procedure. Tubes containing islets were kept in water bath at 37°C for 20 minutes. Every 2–3 minutes, tubes were shaken vigorously and the pancreas gradually dissolved into fine particles. The incubation was terminated by adding 50 mL of medium and centrifuging the tubes at 441 rcf at 4°C for 1 minute. Supernatant was removed, followed by 3 washes. Then the pellet was suspended and transferred to a 15 mL tube, which was centrifuged at 441 rcf for 5 minutes at 4°C. We used Lympholyte 1.1 (Cedarland Lab) to separate islets from the exocrine tissue by centrifuging at 635 rcf for 15 minutes. Islets appeared as a ring between 2 different solutions. The islet layers were pipetted and transferred to two 50 mL tubes, which were centrifuged at 783 rcf for 1 minute at 8°C. This wash was repeated 3 times. Thereafter, islets were resuspended and transferred to a petri dish and incubated at 37°C. Islets were maintained in RPMI 1640 supplemented with 10% FBS and 1% penicillin/streptomycin.

### Cell viability (MTT) and apoptotic (cell death detection) assays.

MIN6 cells were plated at 5 × 10^4^ cells/well in a 96-well plate. Drugs were pretreated for 30 minutes before 24 hours coincubation with 500 μM palmitate or BSA. MTT solution was prepared by dissolving 3-[4,5-dymethylthiazol-2-yl]-2,5-diphenyl tetrazolium bromide in fresh DMEM at 0.5 mg/mL. MTT assay was initiated by replacing culture medium with 100 μL of MTT solution. The plate was put in an incubator during 2 hours. After careful suction of medium to avoid cell detachment, 100 μL of DMSO was pipetted into each well to dissolve the formazan. The plate was then gently swirled for 10 minutes, and absorbance value of each well was measured at 570 nm (reference wavelength 650 nm) by Epoch Microplate Spectrophotometer (Bio-Tek).

The amount of cytoplasmic mono- and oligonucleosome-associated histone-DNA complexes in cell lysates were quantified in a sandwich ELISA (Cell Death Detection ELISA Plus kit, Roche Diagnostics). First, 5 × 10^4^ MIN6 cells/well were seeded into a 96-well plate. Drugs were added for 30 minutes before 24 hours of coincubation with 500 μM palmitate or BSA. The assay was performed by adding 200 μL of lysis buffer supplied in the kit to each well, and the plate was incubated for 30 minutes at room temperature. After centrifugation at 200 rcf for 10 minutes, 20 μL of supernatant was collected, and the following steps were conducted following the protocol of the manufacturer. After 5-minute incubation with a peroxidase substrate, absorbance values were measured at 405 nm (reference wavelength 490 nm) in an Epoch Microplate Spectrophotometer (Bio-Tek).

### Western blot analysis.

For total protein extraction, cells seeded on 6-well plates were washed with ice-cold PBS and lysed with cold RIPA buffer (Thermo Fisher Scientific) containing protease inhibitor cocktail (Roche Diagnostics). The supernatants from lysates were loaded for SDS-PAGE and then transferred to polyvinylidene difluoride membrane (Merck Millipore). The membrane was blocked by 6% skim milk for 1 hour at room temperature, followed by incubation with primary antibody at 4°C overnight. HRP-conjugated secondary antibody (Supplemental Table 1) against either mouse or rabbit IgG was incubated for 1 hour at room temperature. The bands were visualized with a ChemiDoc XRS+ imaging system (Bio-Rad) using ECL solution (Luminata Forte, MilliporeSigma).

### Reverse transcription and real-time PCR.

Total RNA was extracted from MIN6 or islets using Hybrid-R RNA kit. cDNA was synthesized from 0.5–1 g RNA with qPCR RT Master Mix according to the manufacturer’s protocol (Thermo Fisher Scientific). cDNA was amplified using Accupower HotStart PCR premix (Bioneer) to detect the expression of genes of interest. For real-time PCR, each cDNA sample was mixed with SYBR Green PCR Master Mix in a triplicate manner in a real-time PCR machine. ΔΔCt method using *Rn18s* as an internal control was used to analyze relative expression.

### Cytosolic and mitochondrial ROS measurement.

A total of 5 × 10^4^ cells were plated onto 12 mm coverslips embedded in 4-well plate before treatment with palmitate (350 μM, 24 hours) or BSA. Cells were loaded with 2.5 μM CM-H2DCFDA or 5 μM mitoSOX for 10 minutes at 37°C to detect intracellular or mitochondrial ROS, respectively. Excitation/emission wavelengths for DCF and mitoSOX were 490/535 nm and 514/560 nm, respectively. Fluorescence intensity was quantified using the IX81 inverted microscope (EVOS XL Core, Invitrogen) with MetaMorph 6.1.

### mTORC1 activity measurement using TORCAR.

MIN6 cells were plated onto 12 mm coverslips. The next day, cells were transfected with TORCAR plasmid. After 48 hours, MIN6 cells expressed TORCAR and were treated with or without inhibitors. Cells were serum-starved overnight in normal DMEM, then before TORCAR imaging, amino acid–free medium was changed for 2 hours to reduce basal mTORC1 activity. We performed live-cell imaging using an IX-73 inverted microscope (Olympus). During the measurement, cells were stimulated by palmitate (or BSA for vehicle), and the resulting changes in FRET were recorded. Reversed FRET was used to indicate the activation of mTORC1.

### Perilysosomal, ER, and cytosolic Ca^2+^ measurement.

We seeded 3 × 10^4^ cells suspended in 500 μL of complete medium onto 12 mm coverslips embedded in 4-well plates before treatment with palmitate (350 μM, 24 hours) or BSA. Cells were loaded with 1 μM Fura-2 AM dissolved in KRBB for 30 minutes in an incubator. Cells were washed 3 times by KRBB. During recording of the Fura-2 signals, the chamber was perfused with Ca^2+^-free KRBB in the presence of 200 μM sulfinpyrazone unless otherwise mentioned. The chamber was placed on the IX-73 inverted microscope platform attached to a complementary metal-oxide-semiconductor camera and an LED illuminator. The fluorophore was alternately excited at 340 and 380 nm while emission was recorded at 510 nm using MetaFluor 3.1. At the end of each experiment, 10 μM ionomycin was added to induce a maximal ratio for comparison between control and palmitate-treated cells.

To measure ER Ca^2+^ and lysosomal Ca^2+^, G-CEPIA1er (Addgene plasmid #105012) and GCaMP3-TRPML1 provided by Haoxing Xu (University of Michigan, Ann Arbor, Michigan, USA), respectively, were introduced by transfection 48 hours prior to the experiment. The fluorescence emission at 535 nm was captured and subsequently analyzed after excitation at 488 nm.

### Adenovirus.

Recombinant adenoviruses expressing TFEB (Ad/CMV/V5-DEST-TFEB-HA) or an empty backbone (Ad/CMV/V5-DEST) were provided by Abhinav Diwan (Washington University, St. Louis, Missouri, USA). Adenoviruses were amplified in HEK-293A cells (CRL-1573, ATCC), purified using CsCl gradient centrifugation, and titrated using adenovirus titration kit (Agilent).

### Lipid peroxidation assay.

For flow cytometry analysis, cells were incubated with 2 μM BODIPY^581/591^ C11 (Thermo Fisher Scientific, D3861) in culture medium for 30 minutes (37°C) and washed twice with PBS. Fluorescence emission was measured using a flow cytometer (BD Biosciences, FACSAria III), with green fluorescence (oxidized form) detected at 525 nm and red fluorescence (reduced form) at 590 nm. The ratio of green to red fluorescence intensity was calculated to quantify lipid peroxidation levels. Fluorescence images were acquired using a confocal laser scanning microscope (Zeiss LSM 900).

### Immunostaining and confocal microscopy.

MIN6 cells were subjected to transfection with LC3-GFP-RFP or TFEB-GFP constructs. Following a 2-day incubation period, the transfected cells were exposed to either palmitate or BSA, with or without additional drugs. Subsequently, the cells were fixed using 4% paraformaldehyde at room temperature in the absence of light for 15 minutes. Images were acquired utilizing a Zeiss LSM 800 confocal microscope and ZEN2.3 software. To quantify the yellow and red puncta in LC3-RFP-GFP, ImageJ software from NIH was employed. For the assessment of TFEB-GFP localization, the distribution of TFEB in the cytosol or nucleus was quantified.

### Public transcriptome dataset collection and meta-analysis.

A multidisciplinary team of physiologists, data scientists, and clinicians conducted a comprehensive literature and database review to identify candidate datasets, ultimately collecting full-transcriptome profiling datasets from human β cells (NCBI GEO GSE81608, GSE164416, GSE159984, GSE154126). These datasets were standardized through preprocessing, log transformation, and harmonization. Differential expression analysis between diabetes and control groups was performed using DESeq2, followed by a meta-analysis employing the Inverse-Variance Weighted method implemented in the METAL software (http://csg.sph.umich.edu/abecasis/metal/).

### Statistics.

All experiments were conducted with randomized samples and expressed as mean ± SD or SEM. The data analyses were blinded and statistical analysis was undertaken only for studies where number of independent experiments per group was at least 5. Using Prism software (Version 9.1.0.221, GP9-1988787-RLRM-FA1F1, GraphPad Software), unpaired 2-tailed Student’s *t* test or 1-way or 2-way ANOVA was performed. Post hoc Tukey multiple-comparison test after ANOVA was performed only if *F* achieved statistical significance. A *P* value of less than 0.05 was considered statistically significant.

### Study approval.

All animal procedures were approved by the Institutional Animal Care and Use Committee of Yonsei University Wonju College of Medicine (YWC-200907-2). The human islet gene expression analyses used only publicly available, deidentified datasets; therefore, no IRB approval or informed consent was required.

### Data availability.

All underlying data supporting the findings of this study are available as follows: Raw and processed values underlying graphs and reported summary statistics are provided in the accompanying Supporting Data Values file. Code used for analyses is available from GitHub (https://github.com/DMCBaYonsei/TRPML_study-/tree/main/Source_code; commit ID 39bddaf). Public transcriptomic datasets analyzed include GSE81608, GSE164416, GSE159984, and GSE154126.

## Author contributions

HTN, LDL, and KSP performed conceptualization. HTN, SKL, CNP, TL, and KSP developed methodology. HTN, LDL, TTTN, SKL, CNP, XRY, KWC, and SL performed investigation. HTN and KSP wrote the original manuscript draft. HTN, TL, SKC, MSL, AW, CBW, and KSP reviewed and edited the manuscript.

## Funding support

This work is the result of NIH funding, in whole or in part, and is subject to the NIH Public Access Policy. Through acceptance of this federal funding, the NIH has been given a right to make the work publicly available in PubMed Central.

- Medical Research Center Program (2017R1A5A2015369 and RS-2024-00409403) and Bio & Medical Technology Development Program (2020M3A9D8039920 and RS-2024-00397929) of the National Research Foundation of Korea funded by the Korean government.

## Supplementary Material

Supplemental data

Unedited blot and gel images

Supporting data values

## Version 1 11/11/2025

In-Press Preview

## Version 2 12/22/2025

Electronic publication

## Version 3 01/09/2026

Affiliation 3/name correction: Department of Global Medical Science
