# Supplementary material for: Palmitate impairs autophagic degradation via oxidative stress/perilysosomal Ca2+ overload/mTORC1 activation pathway in pancreatic β cells
Source: JCI Insight. 2025 Nov 11;10(24):e192827. doi: 10.1172/jci.insight.192827 (PMC12890490; doi:10.1172/jci.insight.192827)
Supplement: Supplemental data [file jciinsight-10-192827-s236.pdf]

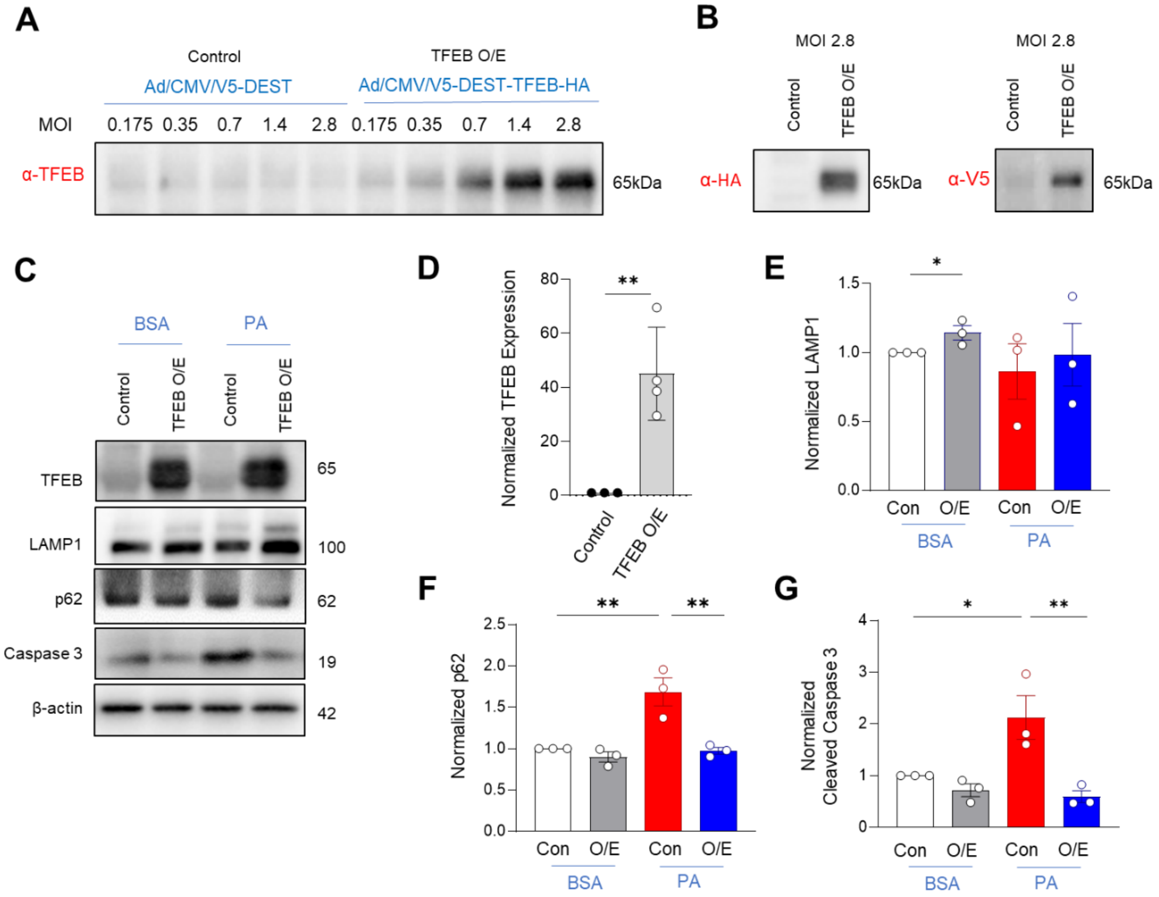

Supplementary Figure S1. Overexpression of TFEB alleviated palmitate-induced autophagy impairment and cytotoxicity. (**A & B**) MIN6 cells were infected with adenoviruses encoding either wild type TFEB (Ad/CMV/V5-DEST-TFEB-HA) or an empty vector control (Ad/CMV/V5-DEST). Expression of TFEB was confirmed by Western blot using anti-TFEB (A), anti-HA (B), and anti-V5 (B). (**C-G**) Overexpression of TFEB upregulated lysosomal protein LAMP1 (E), restored p62 clearance (F), and prevented Caspase-3 activation (G) in palmitate-treated MIN6 cells. Data are presented as means ± standard errors from three independent experiments. Statistical significance was determined using unpaired two-tailed Student's t-test (D) or one-way ANOVA with post-hoc Tukey multiple comparison test (E-G). $^*p<0.05$; $^{**}p<0.01$.

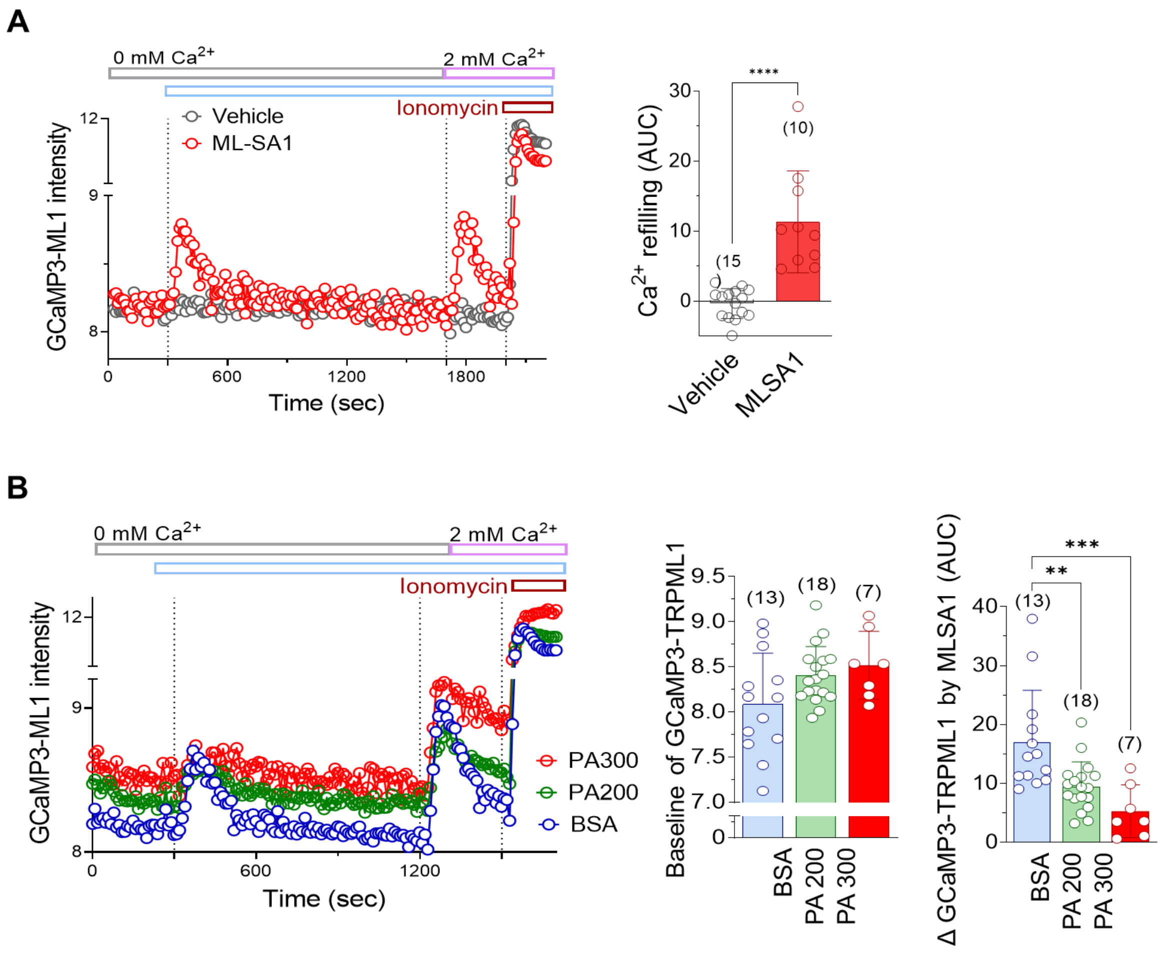

Supplementary Figure S2. Perilysosomal Ca^{2+} measurement using high-throughput fluorescence reader with GCaMP3-TRPML1-expressing cells. (A) Perilysosomal Ca^{2+} rise upon extracellular Ca^{2+} addition, mediated by MLSA1-triggered SOCE. **(B)** Elevated the baseline level of perilysosomal Ca^{2+}, but abolished MLSA1 response by palmitate (PA) in multi-well plates seeded with GCaMP3-TRPML1-expressing HEK cells. Fluorescence intensity from each well were measured by using FlexStation. (n) is the number of analyzed wells from at least 3 independent experiments. Statistical significance was determined using unpaired two-tailed Student's t-test (A) or one-way ANOVA with post-hoc Tukey multiple comparison test (B). $^{**}p<0.01$; $^{***}p<0.001$; $^{****}p<0.0001$.

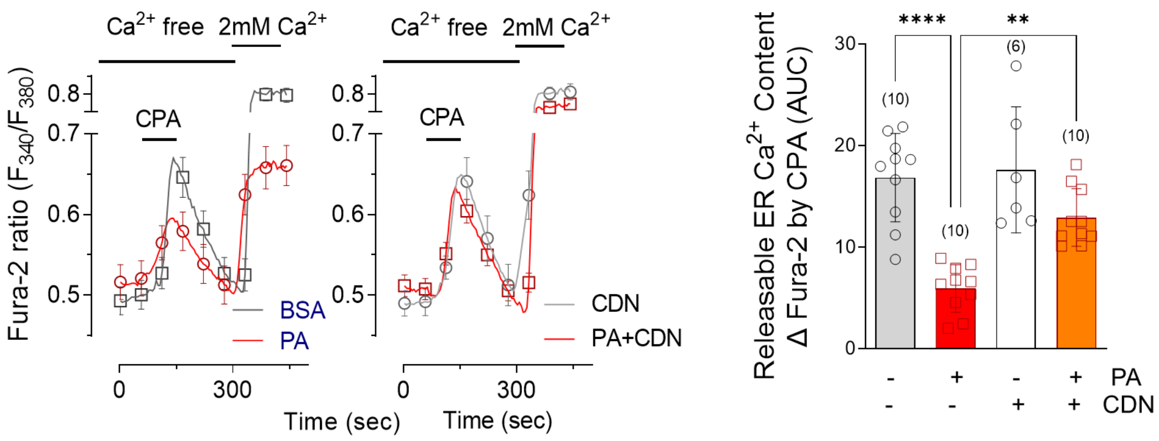

Supplementary Figure S3. SERCA activator recovers ER Ca^{2+} depletion by palmitate. ER Ca^{2+} release due to cyclopiazonic acid (CPA) was reduced in palmitate-treated MIN6 cells, which was normalized by pretreatment with CDN1163, a SERCA activator. Data are presented as means ± standard deviations and (n) is the number of analyzed cells from more than 3 independent experiments. Statistical significance was determined using one-way ANOVA with post-hoc Tukey multiple comparison test. **p<0.01; ****p<0.0001.

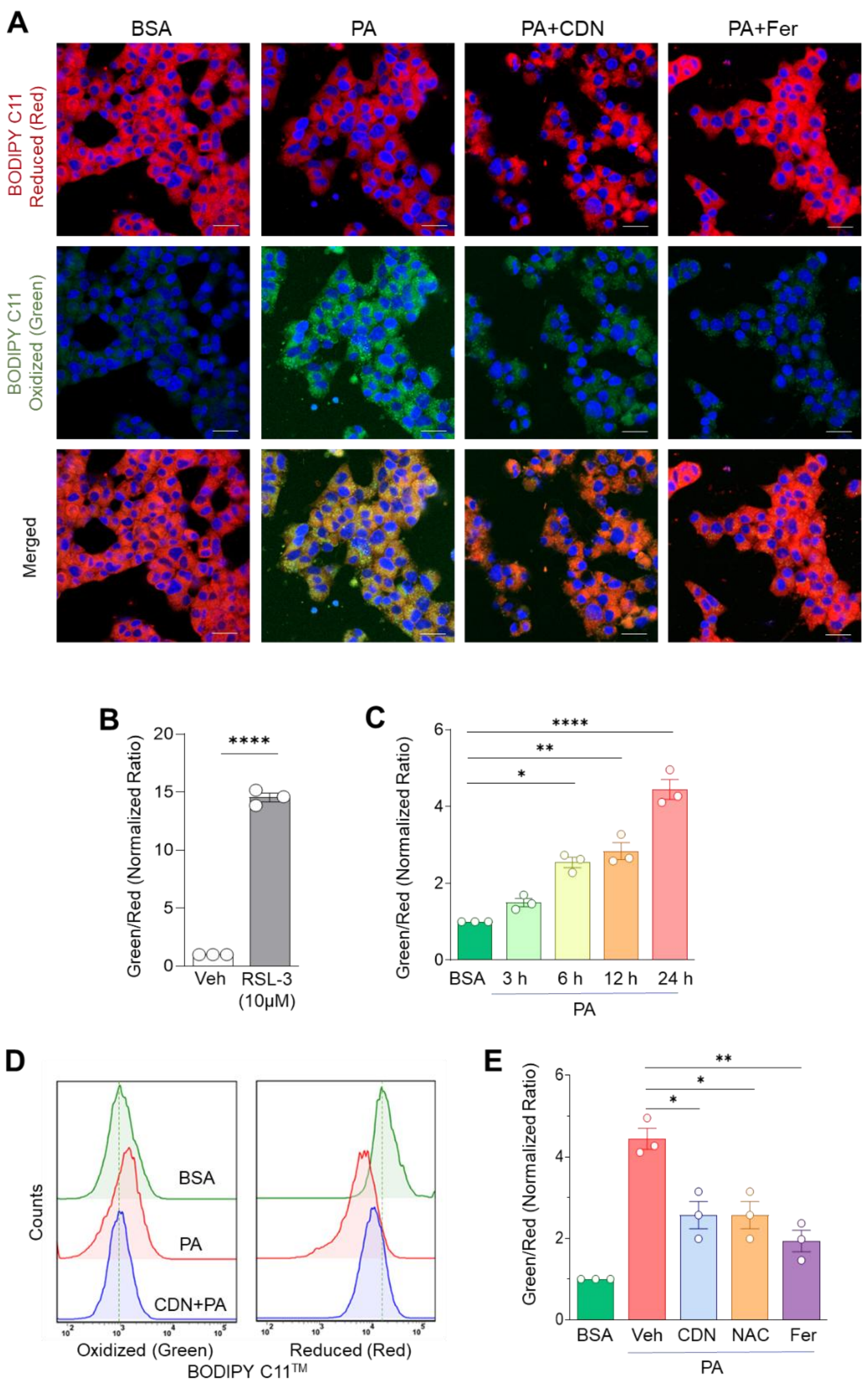

Supplementary Figure S4. SERCA activator and antioxidant mitigate palmitate-induced ferroptotic lipid peroxidation. (**A**) Lipid peroxidation associated with ferroptosis in MIN6 cells was estimated by confocal imaging using BODIPY C11; increased green and decreased red fluorescence indicating ferroptosis. (**B-E**) BODIPY

C11 fluorescence shifts were analyzed by flow cytometry. RSL-3, a ferroptosis inducer through glutathione peroxidase inhibition, increased the green-to-red fluorescence ratio (B). Palmitate also elevated this ratio in a time-dependent manner, suggesting ferroptotic lipid peroxidation. CDN1163, a small-molecule SERCA activator (D & E), and the antioxidant N-acetyl cysteine (NAC; E) reduced palmitate-induced ferroptosis. Additionally, ferrostatin (Fer), a ferroptosis inhibitor, abolished palmitate-induced peroxidation, confirming that the lipid peroxidation was ferroptotic. Data are expressed as means ± standard errors from three independent experiments. Statistical significance was determined using unpaired two-tailed Student's t-test (B) or one-way ANOVA with post-hoc Tukey multiple comparison test (C, E). *$p<0.05$; **$p<0.01$; ****$p<0.0001$.

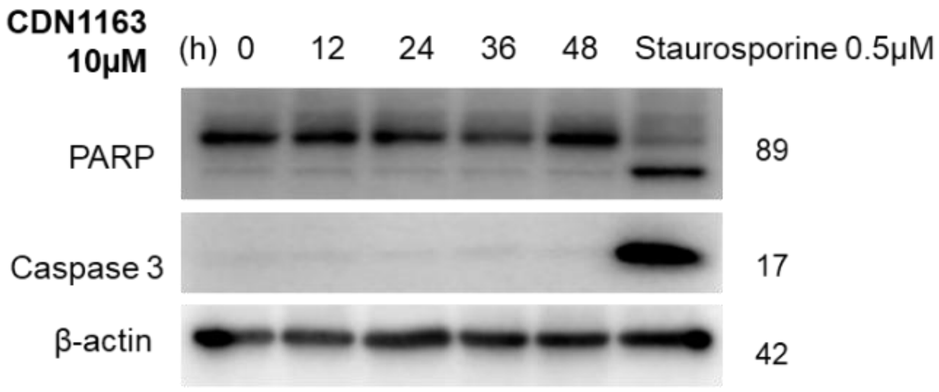

Supplementary Figure S5. CDN1163, a SERCA activator, does not induce cytotoxicity. Cytotoxicity was assessed by detecting PARP cleavage and activated Caspase-3 via Western blot analysis. Incubating MIN6 cells with CDN1163 (10 μM) for 48 h showed no signs of cytotoxicity, whereas treatment with staurosporine (0.5 μM, 8 h) resulted in PARP cleavage and activated Caspase-3.

Supplementary Table 1. Reagents and Tools

Reagents/Resources	Reference/Source	Identifier/Catalogue Number
**Antibodies**		
Rabbit anti-SERCA2	Cell Signaling T.	Cat. 9580, RRID:AB_10827913
Rabbit anti-p62	Cell Signaling T.	Cat.8025, RRID: AB_10562134
Rabbit anti-LC3	Cell Signaling T.	Cat.12741, RRID: AB_2278129
Rabbit anti-p-p70S6K	Cell Signaling T.	Cat.9234, RRID: AB_330944
Rabbit anti-t-p70S6K	Cell Signaling T.	Cat.9202, RRID: AB_330930
Rabbit anti-p-ACC	Cell Signaling T.	Cat.3661, RRID: AB_330337
Rabbit anti-t-ACC	Cell Signaling T.	Cat.3662, RRID: AB_2223182
Rabbit anti-p-AMPK	Cell Signaling T.	Cat.2535, RRID: AB_330330
Rabbit anti-t-AMPK	Cell Signaling T.	Cat.2532, RRID: AB_10622186
Rabbit anti Cleaved Caspase 3	Cell Signaling T.	Cat.9661, RRID: AB_2341188
Rabbit anti-TFEB	Cell Signaling T.	Cat.37785, RRID: AB_2799103
Rabbit anti-LaminA/C	Cell Signaling T.	Cat.2032, RRID: AB_2136278
Mouse anti-β actin	Proteintech	Cat. 20536-1-AP, RRID: AB_10700003
Mouse anti-alpha tubulin	DSHB	Cat# 12G10, RRID: AB_1157911
Rabbit anti-LAMP2	Cell Signaling T.	Cat. 49067, RRID: AB_2798843
Mouse anti-LAMP1	Santa Cruz Bio.	Cat. 20011, RRID: AB_626853
Rabbit anti-TFEB	Proteintech	Cat. 13372-1, RRID: AB_2199611
Mouse anti-V5-Tag	Invitrogen	Cat. R960-25, RRID: AB_2556564
Rabbit anti-HA-Tag	Cell Signaling T.	Cat. 3724, RRID:AB_1549585
Rabbit anti-PARP	Cell Signaling T.	Cat. 9542, RRID:AB_2160739
**Chemicals, Reagents**		
CDN1163	Sigma Aldrich	Cat. SML1682
Torin-1	Sigma Aldrich	Cat. 475991
CM-H2DCFDA	Invitrogen	Cat. C6827
mitoSOX	Invitrogen	Cat. M36008
GPN	Santa Cruz Biotechnology	Cat. G6149
MLSA1	Sigma Aldrich	Cat. SML0627
Ionomycin	Alomone labs	Cat. I-700
Menadione	Sigma Aldrich	Cat. M5625

H_2O_2	Sigma Aldrich	Cat. H1009
MitoTEMPO	Sigma Aldrich	Cat. SML0737
BAPTA-AM	Sigma Aldrich	Cat. A1076
W7	Sigma Aldrich	Cat. A4844
KN62	Sigma Aldrich	Cat. K1125
Verapamil	Sigma Aldrich	Cat. V4629
Nimodipine	Sigma Aldrich	Cat. N149
Diazoxide	Sigma Aldrich	Cat. D9035
Fura-2 AM	Invitrogen	Cat. IN-F1201
JC-1	Invitrogen	Cat. T3168
Cyclopiazonic acid (CPA)	Sigma Aldrich	Cat. C1530
Oligomycin	Sigma Aldrich	Cat. 75351
FCCP	Sigma Aldrich	Cat. C2920
Rotenone	Sigma Aldrich	Cat. R8875
Anitmycin A	Sigma Aldrich	Cat. A8674
Paraformaldehyde	Sigma Aldrich	Cat. P6148
Sodium Palmitate	Sigma Aldrich	Cat. P9767
Bovine serum albumin	Sigma Aldrich	Cat. A6003
DAPI Fluoromount-G	Roche	Cat. 10236276001
Ribo EX	GeneAll	Cat. 301-001
Beta-mercaptoethanol	Sigma Aldrich	Cat. M6250
RIPA buffer	SupremeTech	Cat. ST1090-RI
Phosphatase inhibitor (PhosSTOP)	Roche	Cat. 4906845001
Protease inhibitor	Roche	Cat. 5892791001
X-tremeGENE HP DNA Transfection Reagent	Roche	Cat. 6366236001
OptiMEM I	Thermo Fisher Scientific	Cat. 31985-062
BODIPYTM 581/591 C11	Thermo Fisher Scientific	Cat.D3861
**Critical commercial assays**		
MTT	Sigma Aldrich	Cat. M2128
Cell Death Detection ELISA kit	Roche	Cat. 11774425001
Adeno-X^{TM} Rapid Titer kit	Takara	Cat. 632250
**Experimental models**		
MIN6	AddexBio	RRID:CVCL_0431
C57BL/6N	DBL	
**Software and algorithms**		
Prism 9.1	GraphPad	GP9-1988787-RLRM-

		FA1F1
Image J		
**Others**		
siATP2a2	Dharmacon	Cat. M040968-02-0050
Human SERCA2a (pcDNA3.1+)	Addgene	Cat. 75187
Human SERCA2b (pcDNA3.1+)	Addgene	Cat. 75188
pCAG G-CEPIA1er	Addgene	Cat. 105012
2mt-CEPIA plasmid	Addgene	Cat. 58218
GCaMP3-TRPML1	from Prof. Xu	
LC3-GFP-RFP	Addgene	Cat. 21074
TFEB-pEGFP	Addgene	Cat. 38119
pcDNA3-Lyso-TORCAR	Addgene	Cat. 64929
