# Supplementary material for: Palmitate impairs autophagic degradation via oxidative stress/perilysosomal Ca2+ overload/mTORC1 activation pathway in pancreatic β cells
Source: JCI Insight. 2025 Nov 11;10(24):e192827. doi: 10.1172/jci.insight.192827 (PMC12890490; doi:10.1172/jci.insight.192827)
Supplement: Unedited blot and gel images [file jciinsight-10-192827-s237.pdf]

Figure 1B

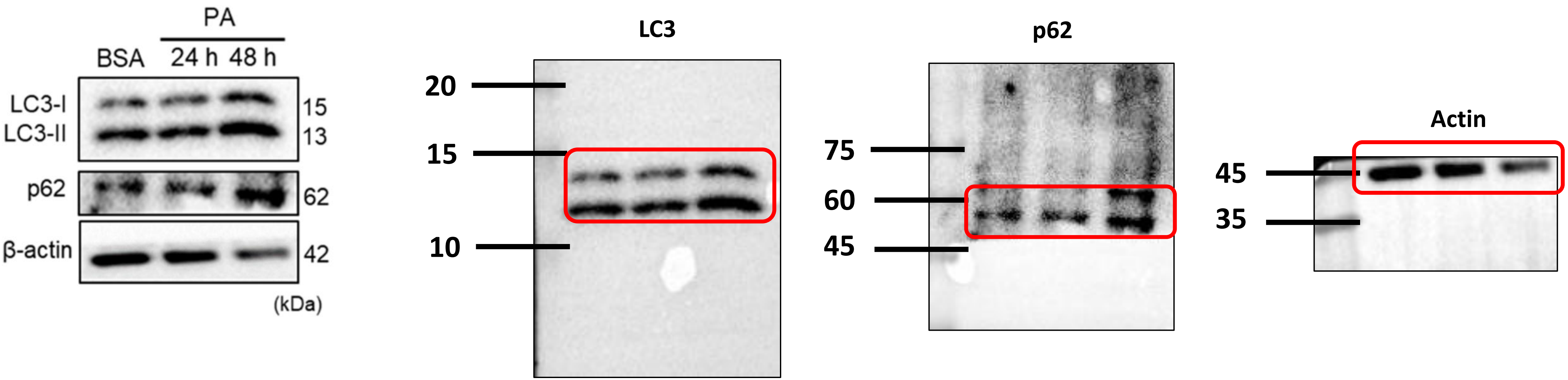

PA
BSA
24 h
48 h
LC3-I
LC3-II
p62
β-actin
15
13
62
42
(kDa)
LC3
20
15
10
p62
75
60
45
Actin
45
35

Figure 1D

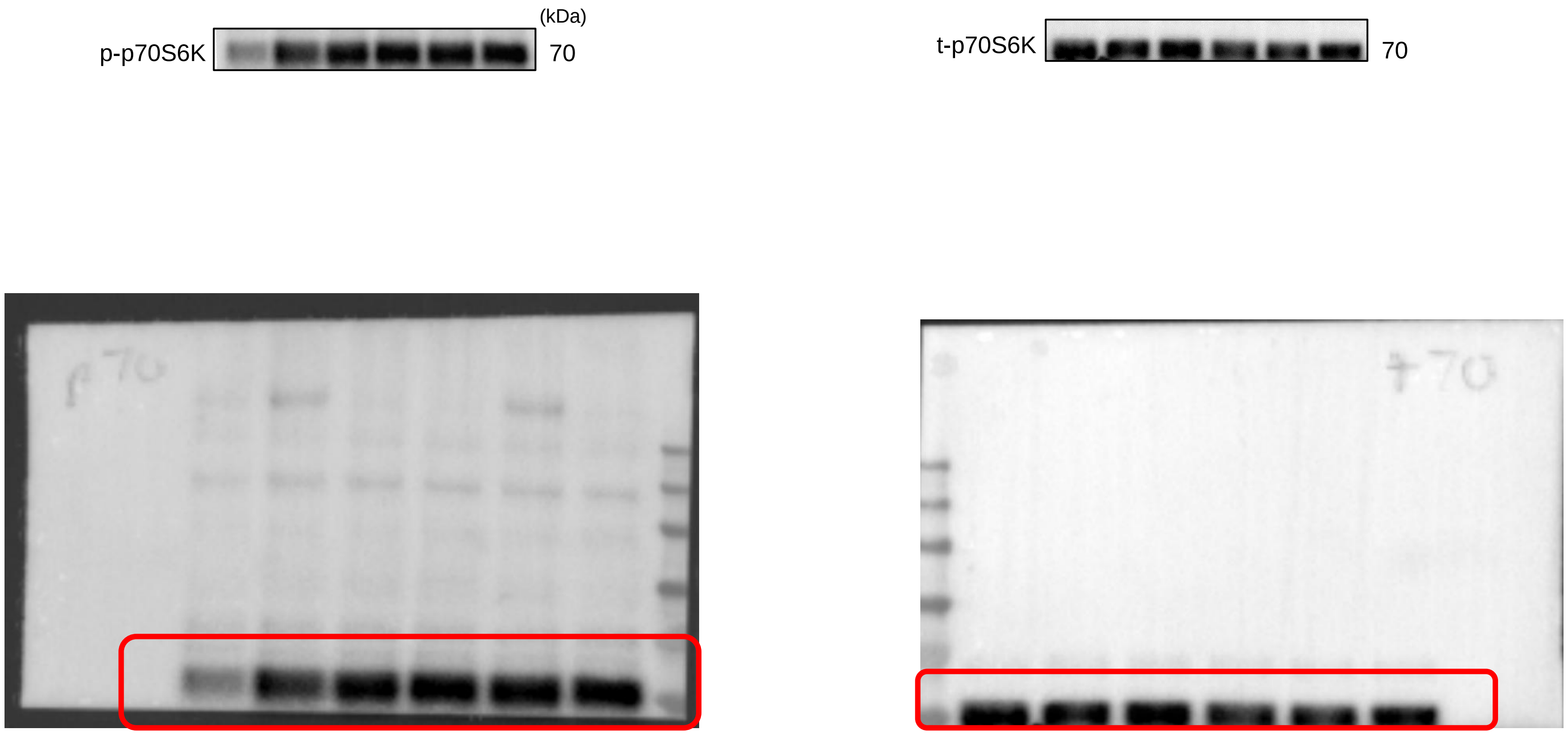

Figure 1D

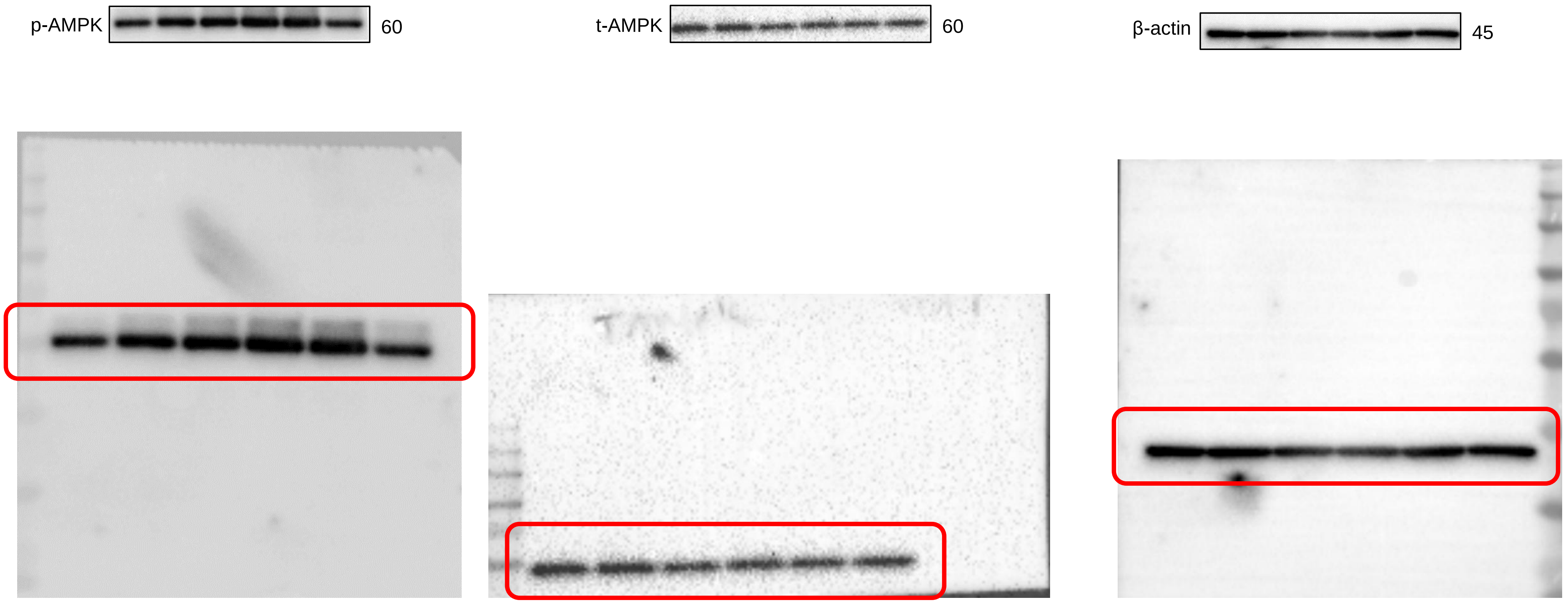

Figure 1H

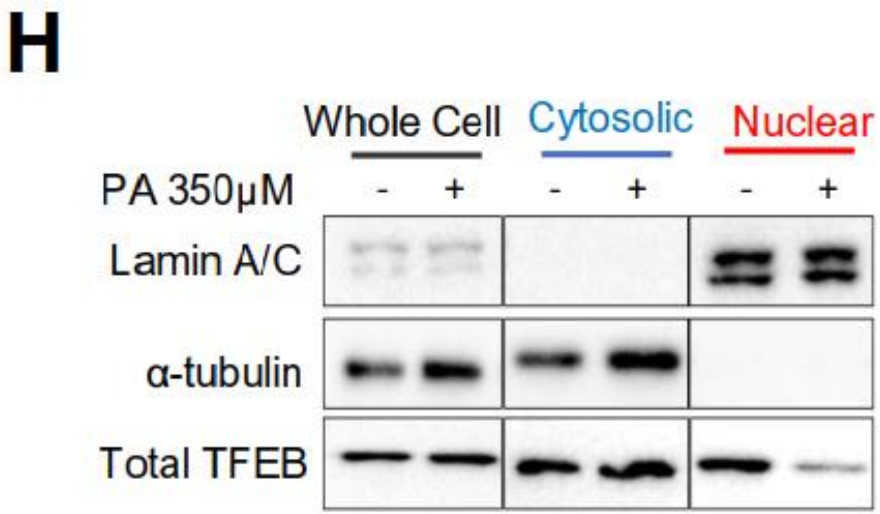
H
Whole Cell
Cytosolic
Nuclear
PA 350μM
- + - + - +
Lamin A/C
α-tubulin
Total TFEB

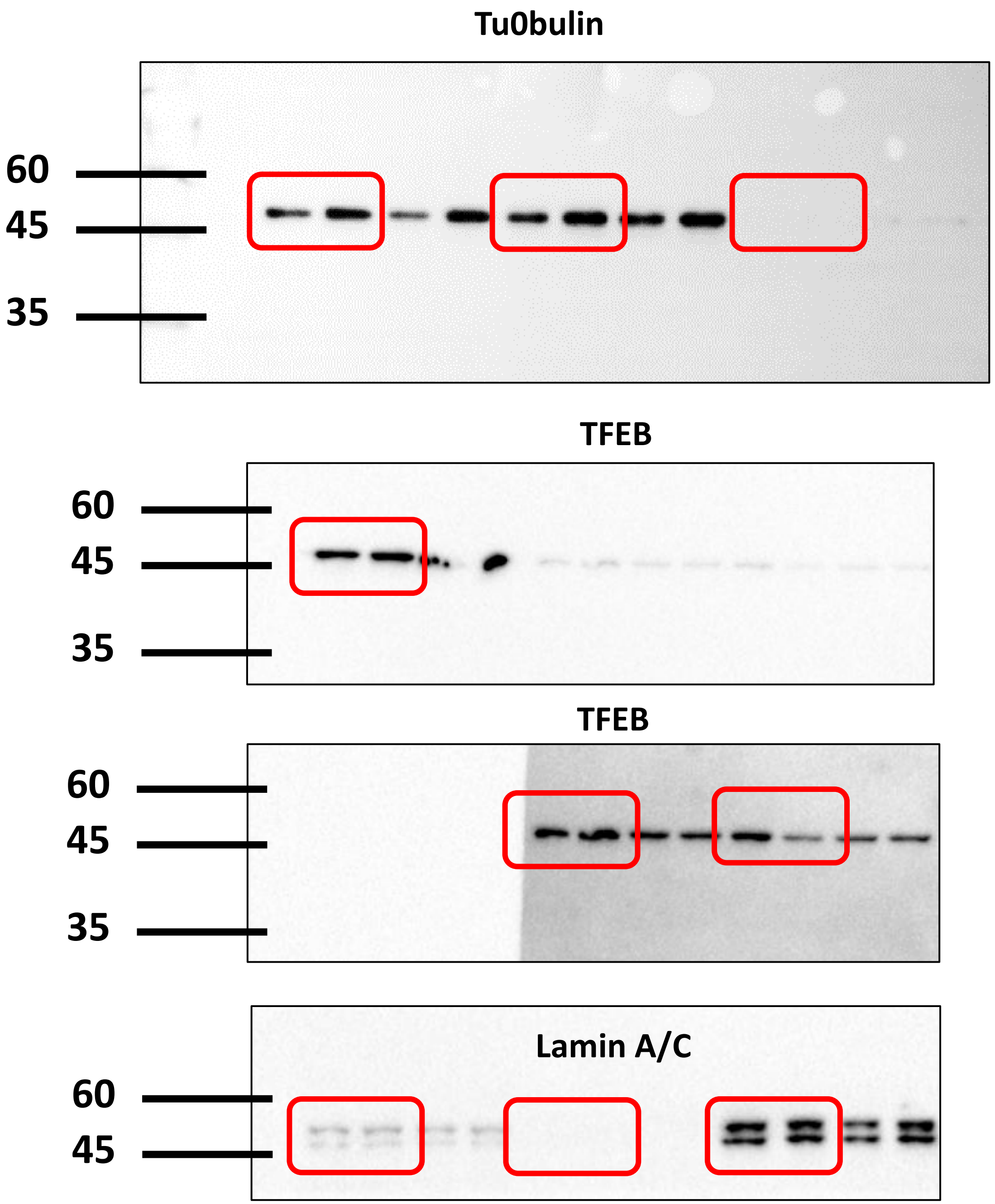
Tu0bulin
60
45
35
TFEB
60
45
35
TFEB
60
45
35
Lamin A/C
60
45

Figure 1J

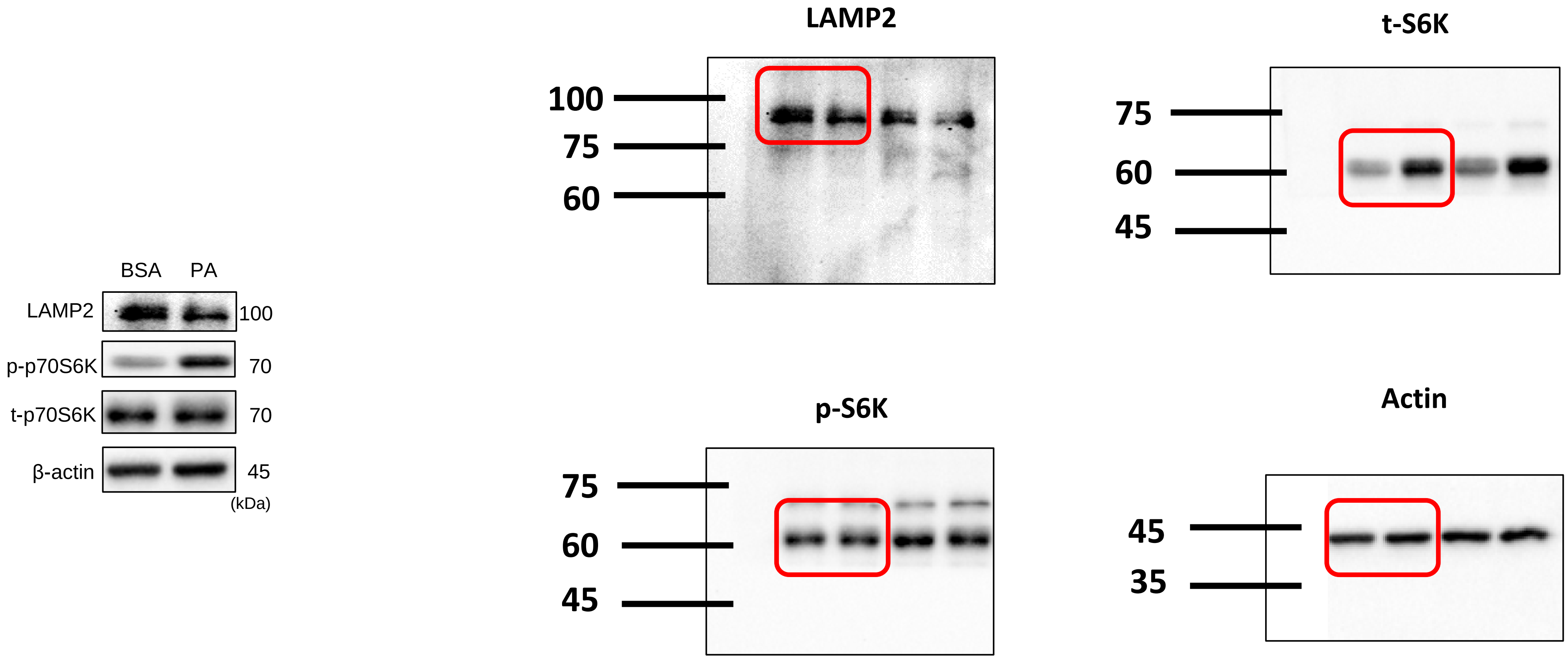
BSA
PA
LAMP2
p-p70S6K
t-p70S6K
β-actin
100
70
70
45
(kDa)
LAMP2
100
75
60
t-S6K
75
60
45
p-S6K
75
60
45
Actin
45
35

Figure 3A

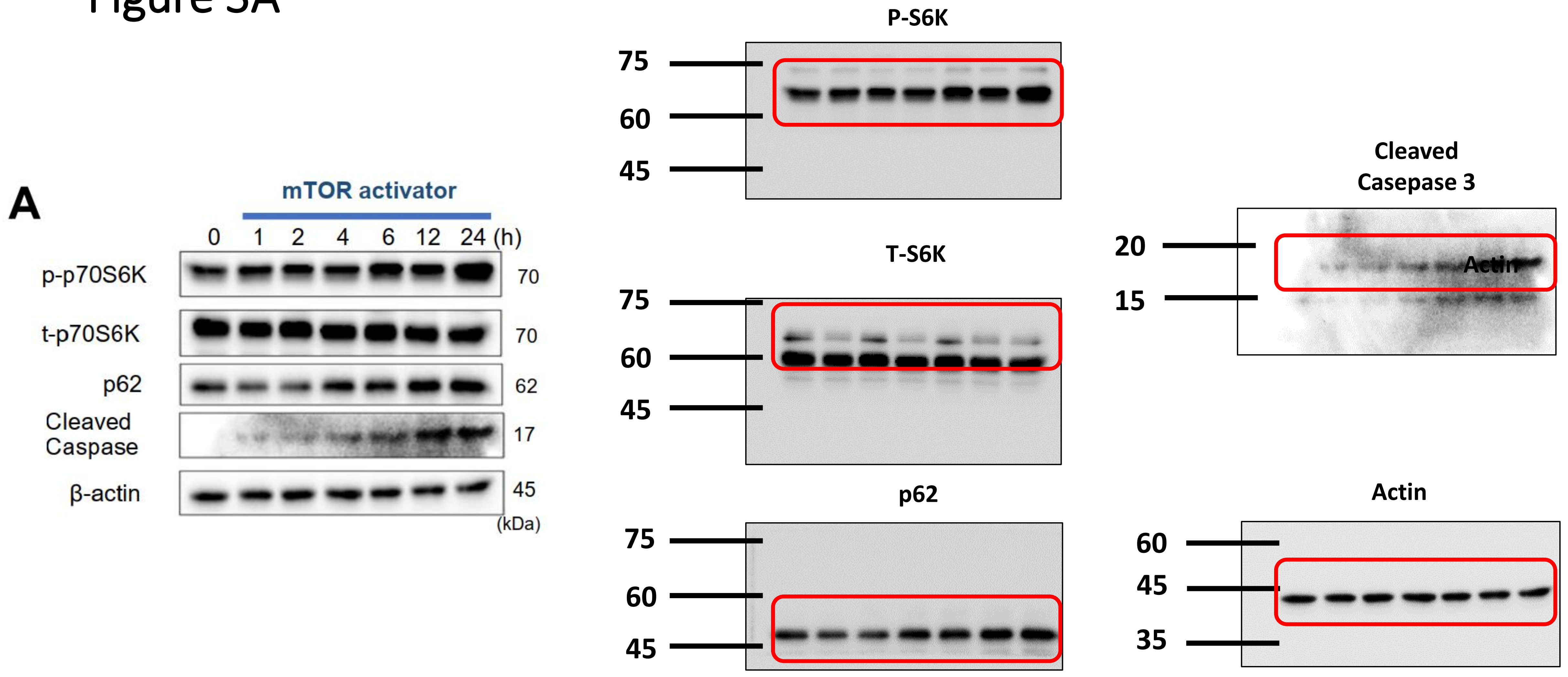

A
mTOR activator
0 1 2 4 6 12 24 (h)
p-p70S6K
t-p70S6K
p62
Cleaved Caspase
β-actin
70
70
62
17
45
(kDa)
P-S6K
75
60
45
T-S6K
75
60
45
p62
75
60
45
Cleaved Casepase 3
20
15
Actin
Actin
60
45
35

Figure 3G

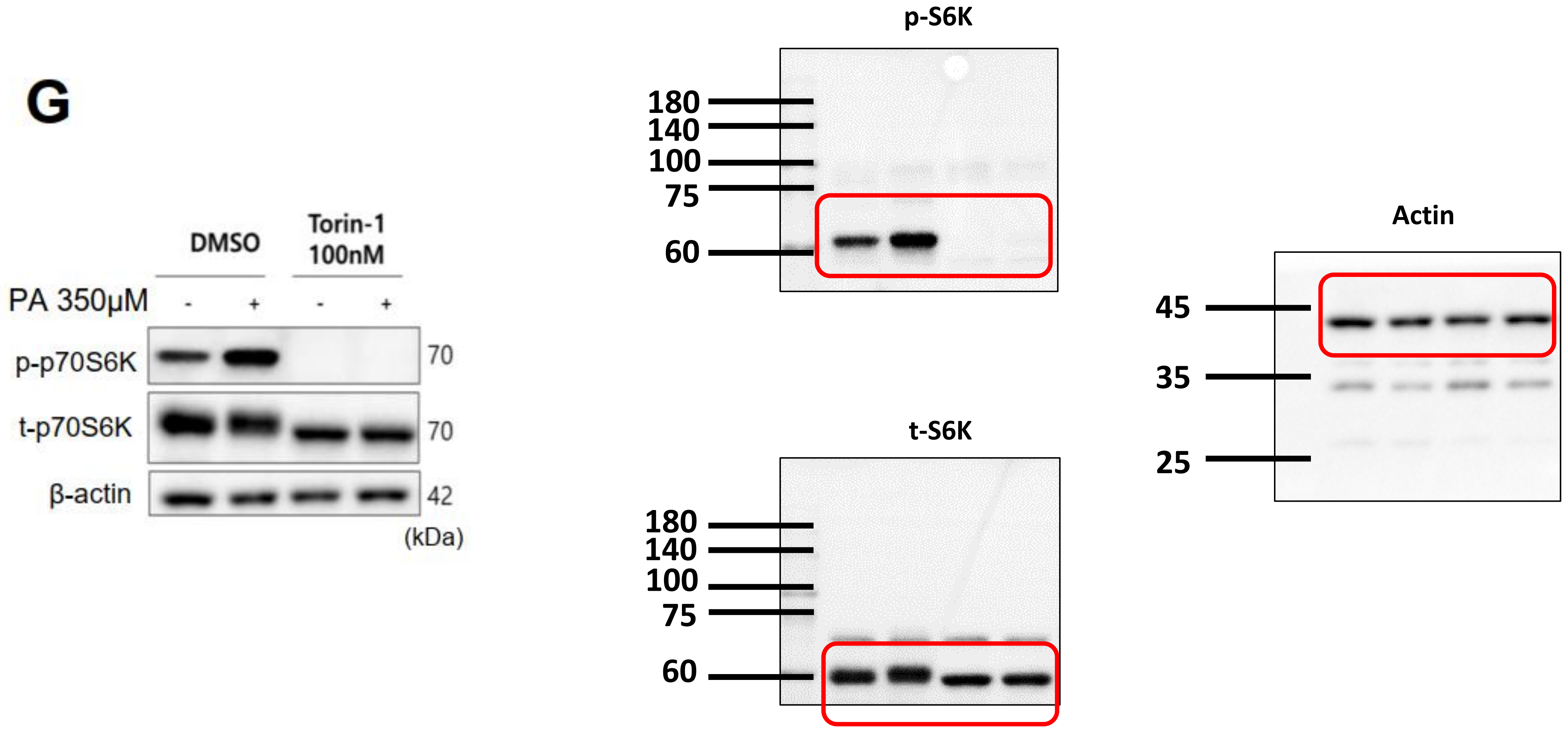
G
DMSO
Torin-1 100nM
PA 350μM
- + - +
p-p70S6K
t-p70S6K
β-actin
70
70
42
(kDa)
p-S6K
180
140
100
75
60
t-S6K
180
140
100
75
60
Actin
45
35
25

Figure 4A

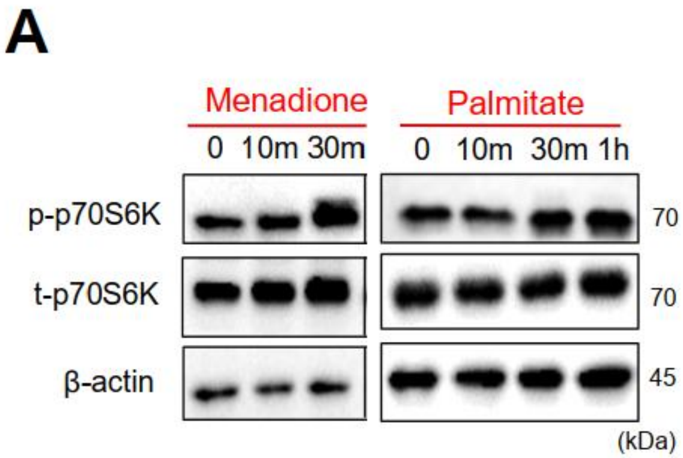
A
Menadione
Palmitate
0 10m 30m
0 10m 30m 1h
p-p70S6K
t-p70S6K
β-actin
70
70
45
(kDa)

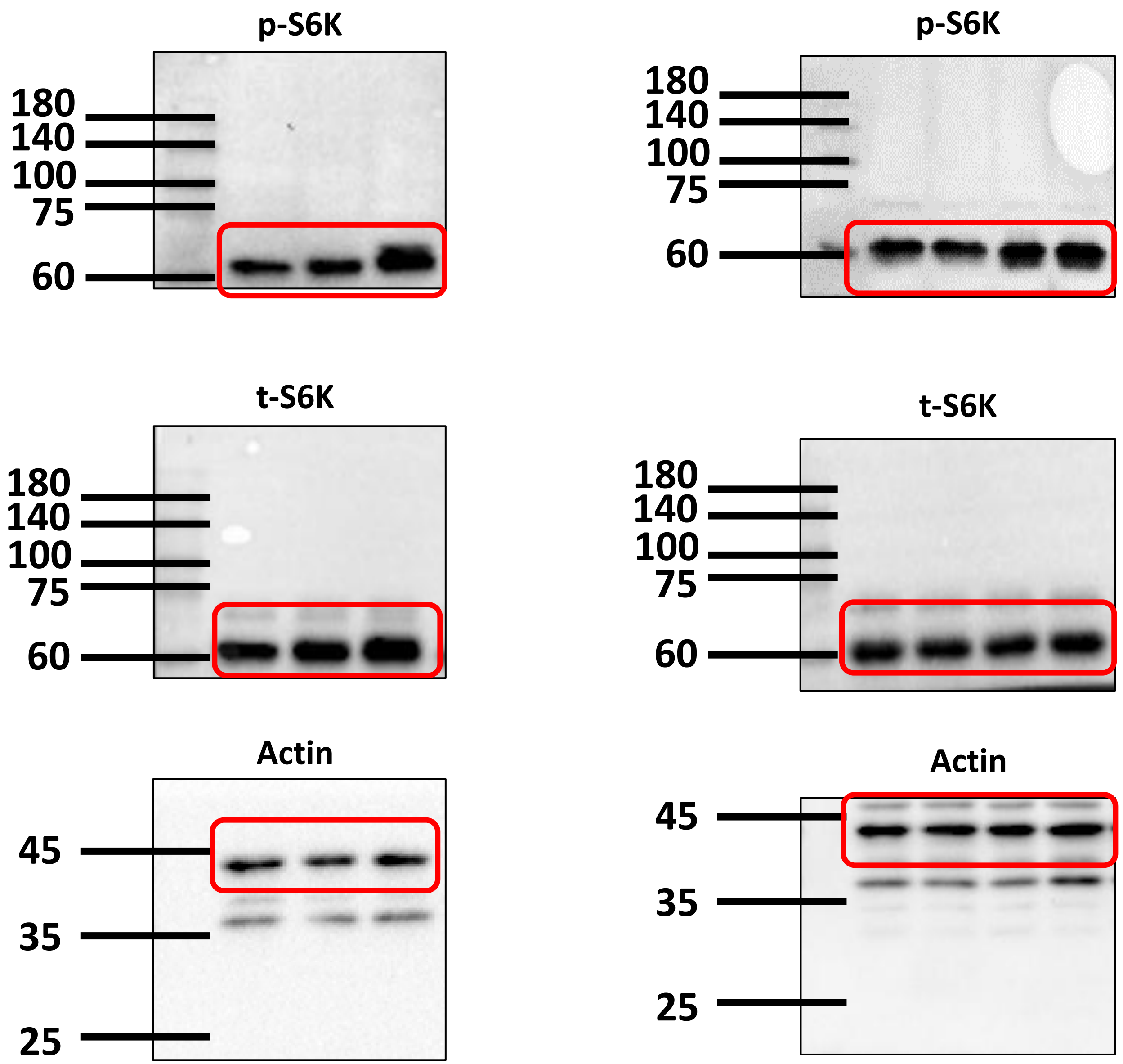
p-S6K
180
140
100
75
60
p-S6K
180
140
100
75
60
t-S6K
180
140
100
75
60
t-S6K
180
140
100
75
60
Actin
45
35
25
Actin
45
35
25

Figure 4E

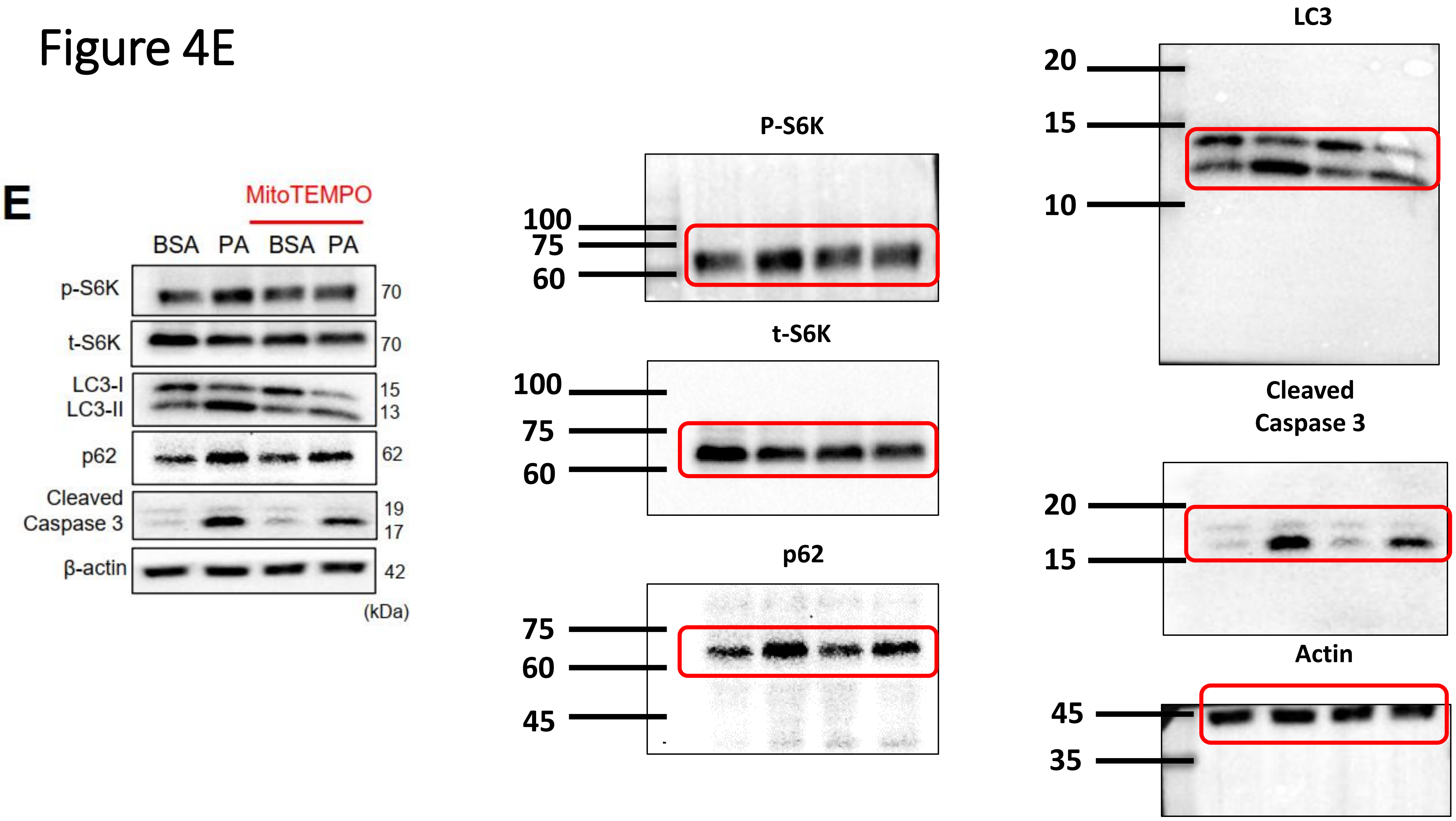
E
MitoTEMPO
BSA PA BSA PA
p-S6K 70
t-S6K 70
LC3-I 15
LC3-II 13
p62 62
Cleaved Caspase 3 19 17
β-actin 42
(kDa)
P-S6K
100
75
60
t-S6K
100
75
60
p62
75
60
45
LC3
20
15
10
Cleaved Caspase 3
20
15
Actin
45
35

Figure 5A

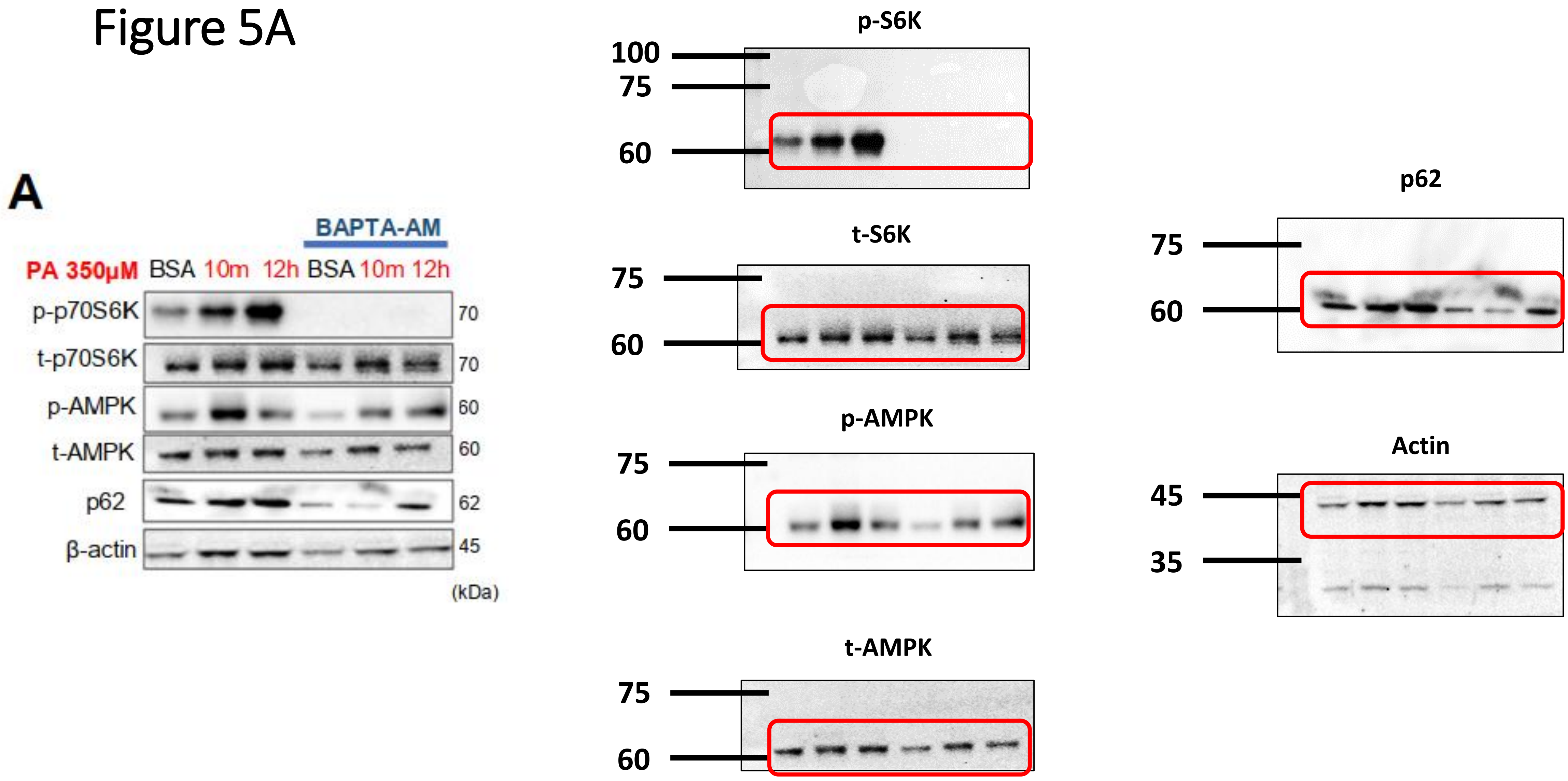

A
BAPTA-AM
PA 350μM BSA 10m 12h BSA 10m 12h
p-p70S6K 70
t-p70S6K 70
p-AMPK 60
t-AMPK 60
p62 62
β-actin 45
(kDa)
p-S6K
100
75
60
t-S6K
75
60
p-AMPK
75
60
t-AMPK
75
60
p62
75
60
Actin
45
35

Figure 5B

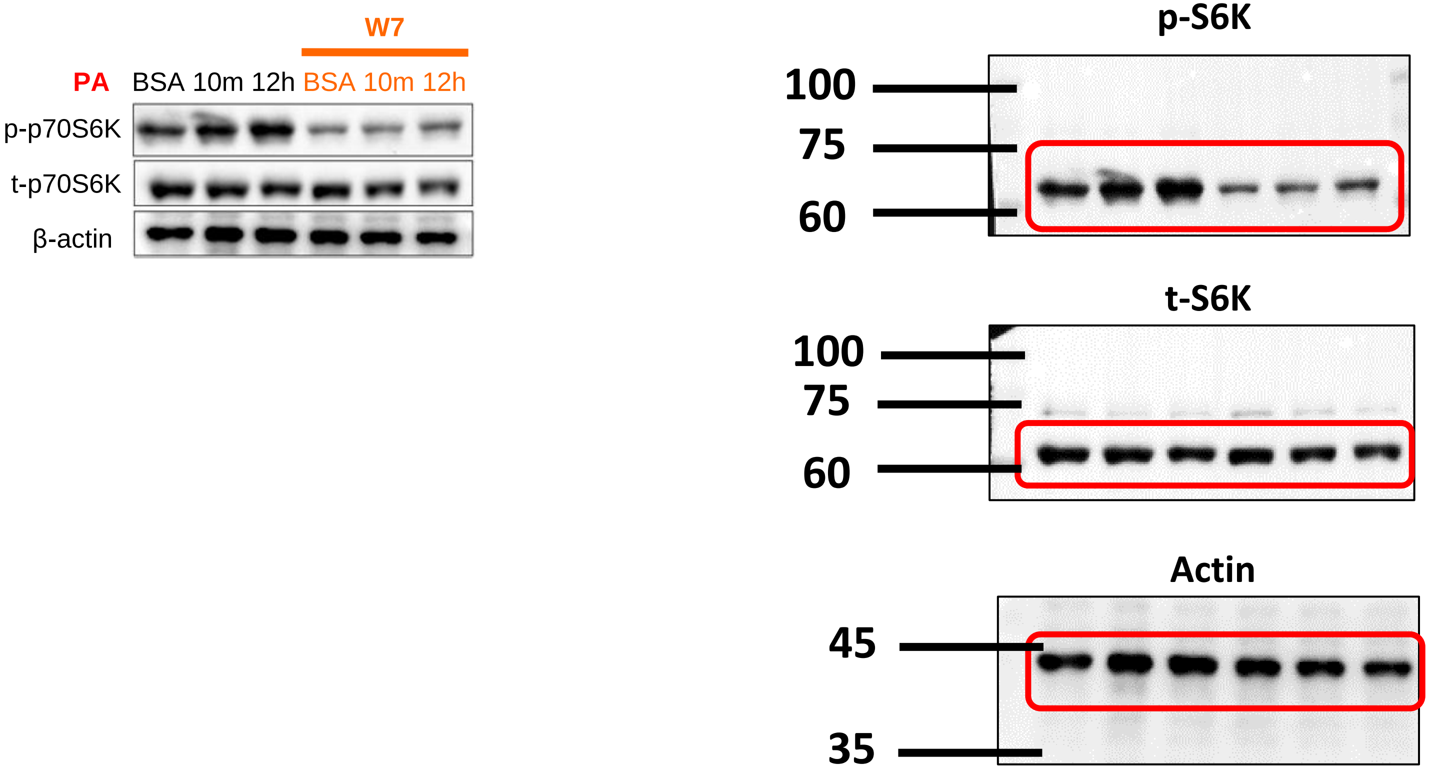
W7
PA BSA 10m 12h BSA 10m 12h
p-p70S6K
t-p70S6K
β-actin
p-S6K
100
75
60
t-S6K
100
75
60
Actin
45
35

Figure 5B-KN62

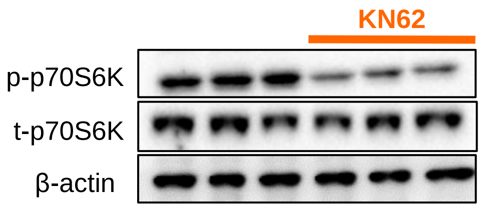

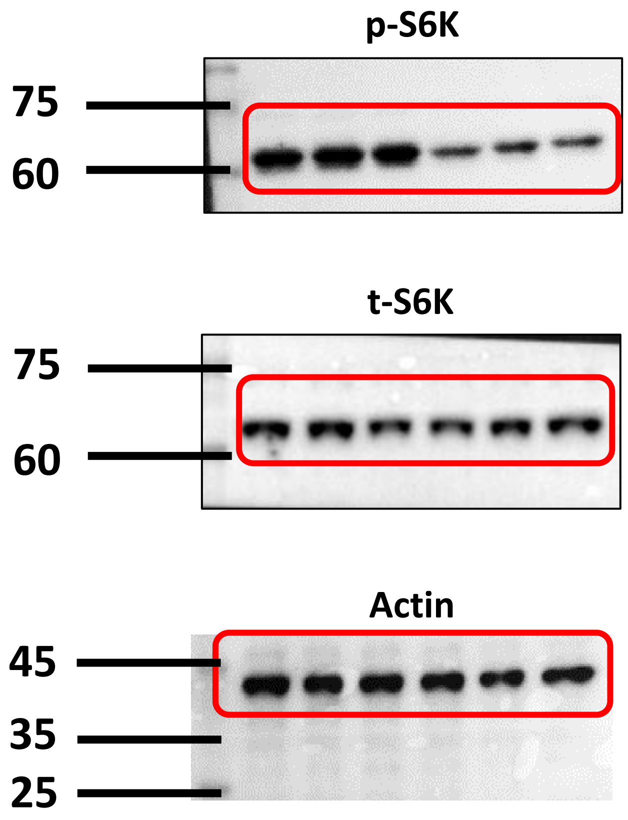

Figure 5D

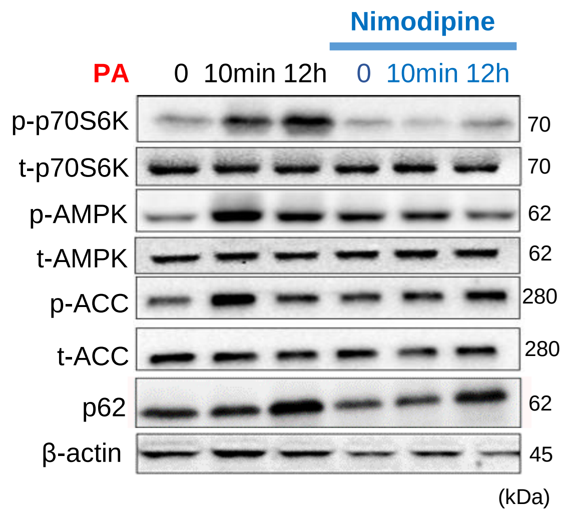

Nimodipine
PA 0 10min 12h 0 10min 12h
p-p70S6K 70
t-p70S6K 70
p-AMPK 62
t-AMPK 62
p-ACC 280
t-ACC 280
p62 62
β-actin 45
(kDa)

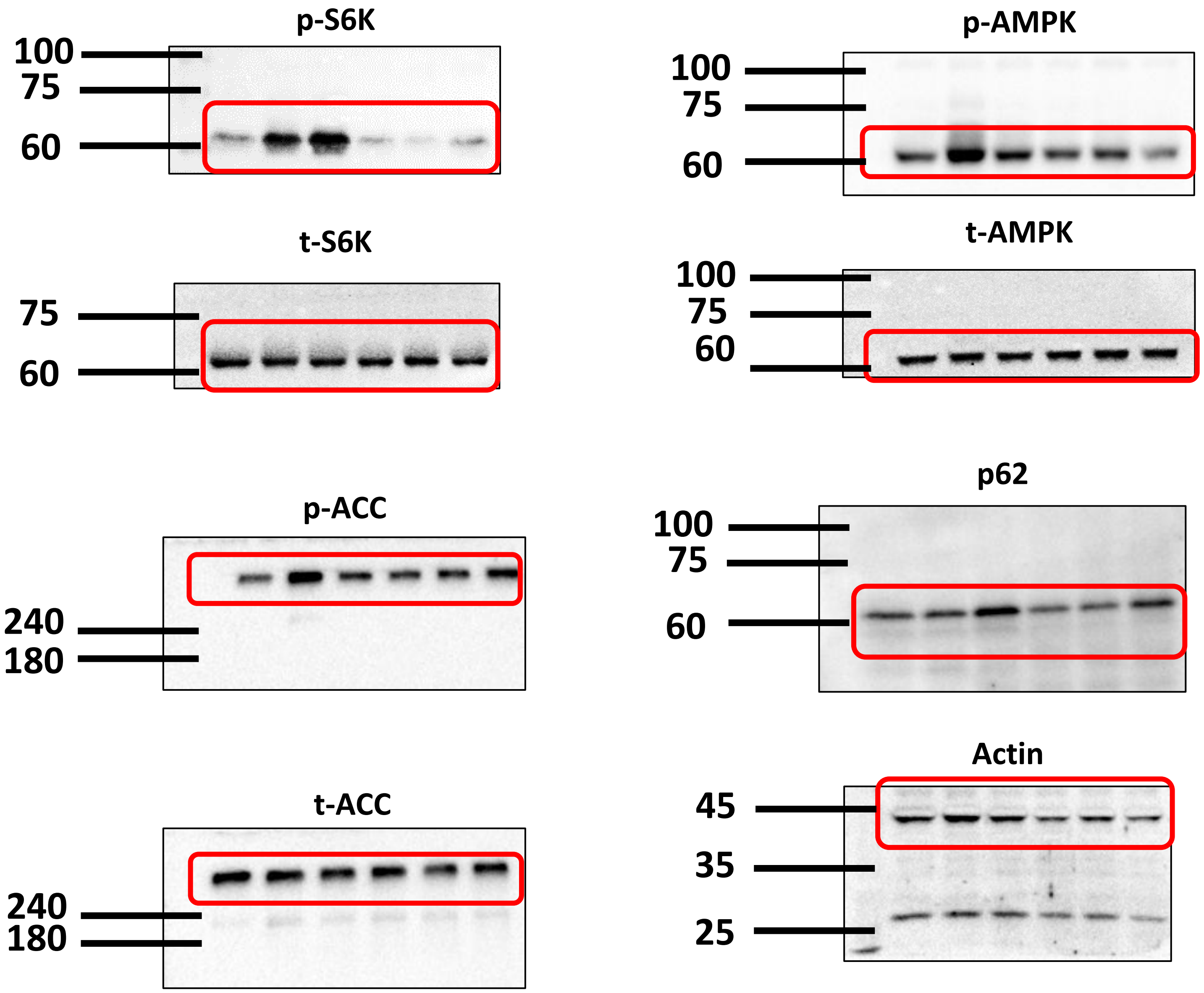

p-S6K
100
75
60
t-S6K
75
60
p-ACC
240
180
t-ACC
240
180
p-AMPK
100
75
60
t-AMPK
100
75
60
p62
100
75
60
Actin
45
35
25

Figure 5E

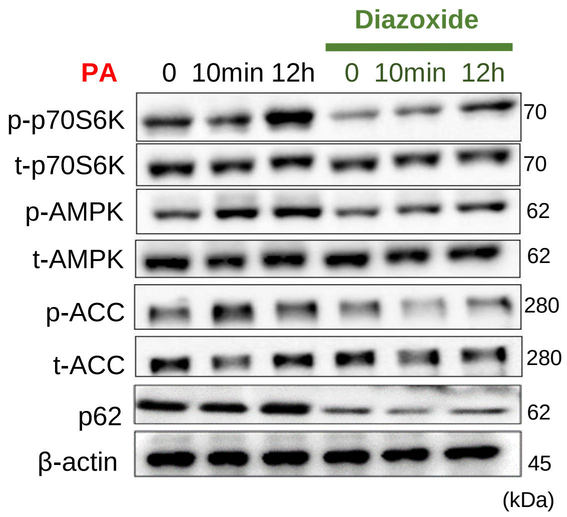

Diazoxide
PA
0 10min 12h
0 10min 12h
p-p70S6K
t-p70S6K
p-AMPK
t-AMPK
p-ACC
t-ACC
p62
β-actin
70
70
62
62
280
280
62
45
(kDa)

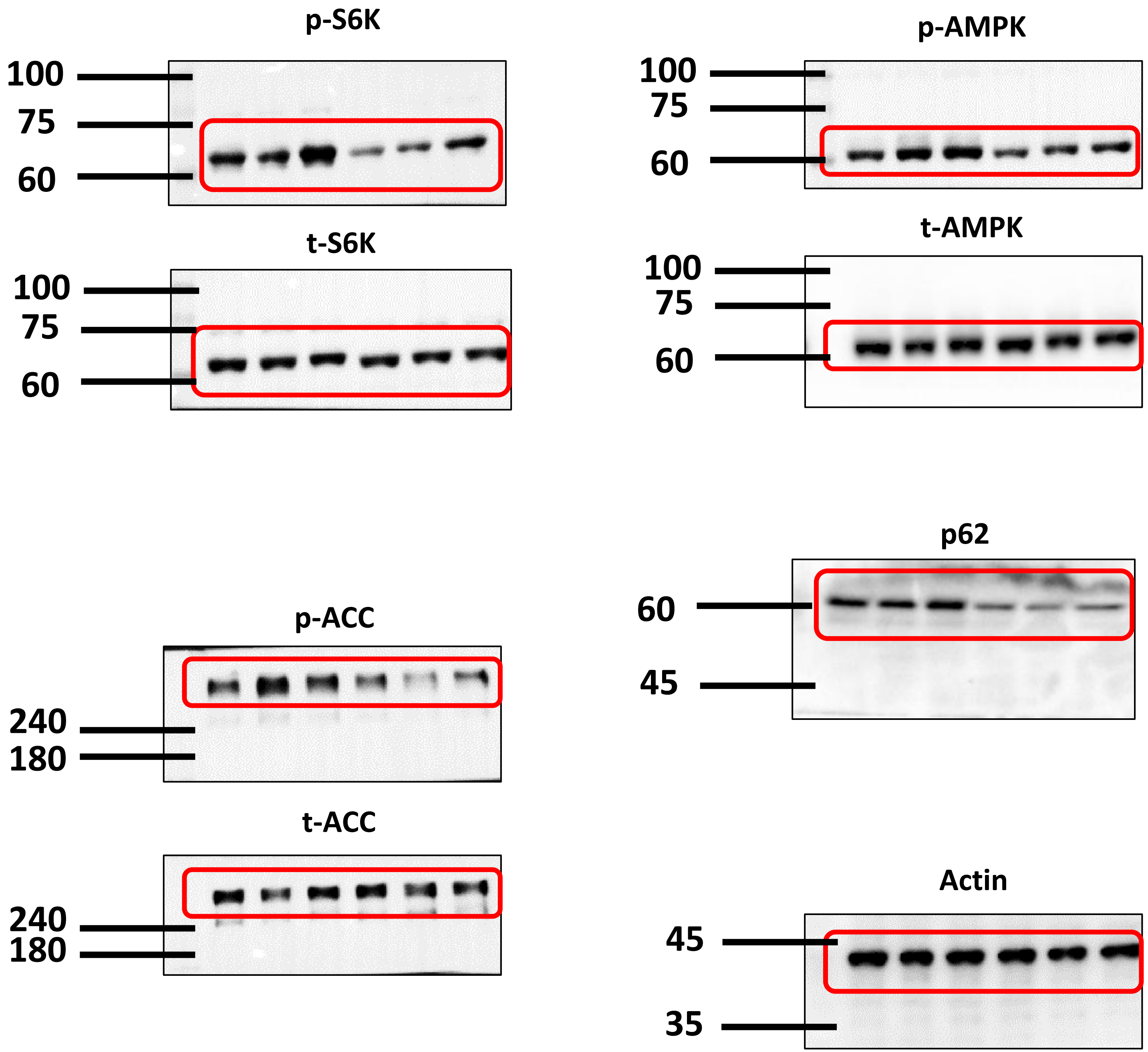

p-S6K
100
75
60
t-S6K
100
75
60
p-ACC
240
180
t-ACC
240
180
p-AMPK
100
75
60
t-AMPK
100
75
60
p62
60
45
Actin
45
35

Figure 6E

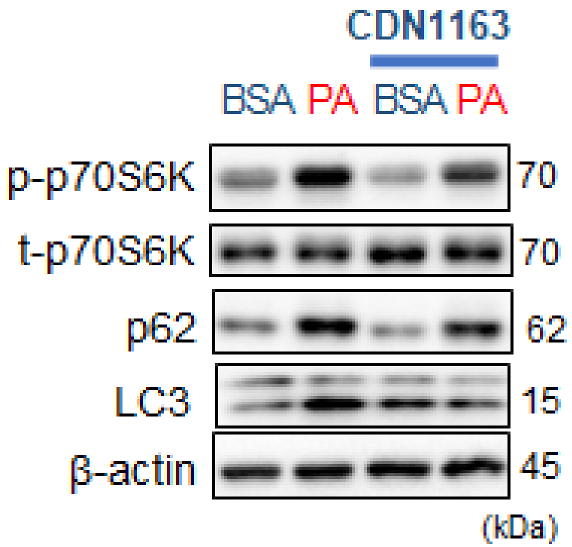

CDN1163
BSA PA BSA PA
p-p70S6K
70
t-p70S6K
70
p62
62
LC3
15
β-actin
45
(kDa)

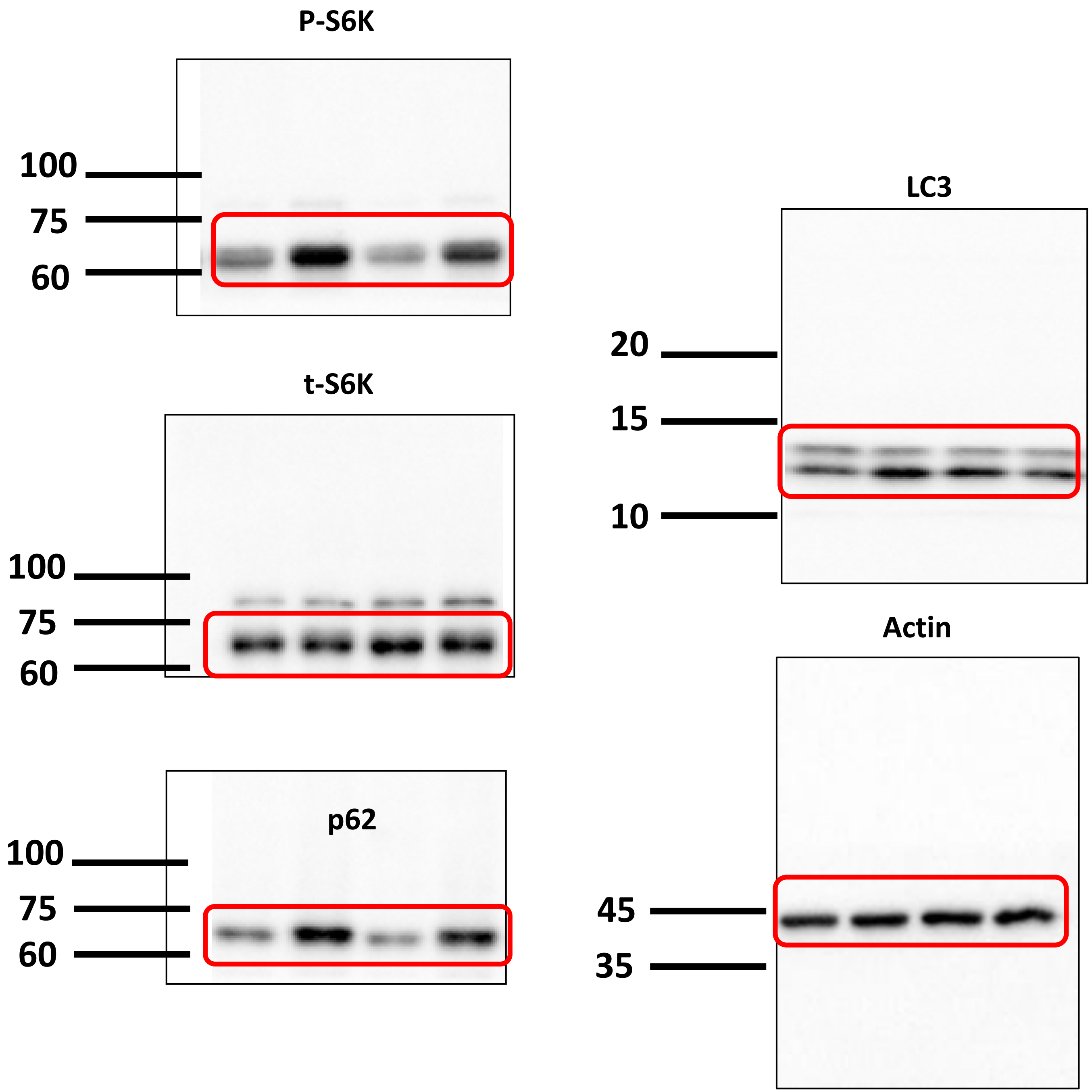

P-S6K
100
75
60
t-S6K
100
75
60
p62
100
75
60
LC3
20
15
10
Actin
45
35
